# Genomics and epigenetics guided identification of tissue-specific genomic safe harbors

**DOI:** 10.1186/s13059-022-02770-3

**Published:** 2022-09-21

**Authors:** Dewan Shrestha, Aishee Bag, Ruiqiong Wu, Yeting Zhang, Xing Tang, Qian Qi, Jinchuan Xing, Yong Cheng

**Affiliations:** 1 Department of Genetics, Genomics, and Informatics, College of Graduate Health Sciences, The University of Tennessee Health Science Center, Memphis, TN USA; 2 Department of Hematology, St. Jude Children’s Research Hospital, Memphis, TN USA; 3 Department of Genetics, Rutgers, The State University of New Jersey, Piscataway, NJ USA; 4 Human Genetics Institute of New Jersey, Rutgers, the State University of New Jersey, Piscataway, NJ USA; 5 Department of Computational Biology, St. Jude Children’s Research Hospital, Memphis, TN USA

**Keywords:** Genomic safe harbor, Genetic engineering, Gene therapy, Epigenome, Chromatin organization, Mobile genetic elements

## Abstract

**Background:**

Genomic safe harbors are regions of the genome that can maintain transgene expression without disrupting the function of host cells. Genomic safe harbors play an increasingly important role in improving the efficiency and safety of genome engineering. However, limited safe harbors have been identified.

**Results:**

Here, we develop a framework to facilitate searches for genomic safe harbors by integrating information from polymorphic mobile element insertions that naturally occur in human populations, epigenomic signatures, and 3D chromatin organization. By applying our framework to polymorphic mobile element insertions identified in the 1000 Genomes project and the Genotype-Tissue Expression (GTEx) project, we identify 19 candidate safe harbors in blood cells and 5 in brain cells. For three candidate sites in blood, we demonstrate the stable expression of transgene without disrupting nearby genes in host erythroid cells. We also develop a computer program, Genomics and Epigenetic Guided Safe Harbor mapper (GEG-SH mapper), for knowledge-based tissue-specific genomic safe harbor selection.

**Conclusions:**

Our study provides a new knowledge-based framework to identify tissue-specific genomic safe harbors. In combination with the fast-growing genome engineering technologies, our approach has the potential to improve the overall safety and efficiency of gene and cell-based therapy in the near future.

**Supplementary Information:**

The online version contains supplementary material available at 10.1186/s13059-022-02770-3.

## Background

Gene and cell-based therapies usually rely on stable expression of transgene to replace defective genes [1, 2], enhance cell functions [3], and improve the safety of engineered cells [4, 5]. However, most of the transgenes are delivered with lentivirus/retrovirus vectors and integrated into the genome in a random or semi-random manner [6], leading to unpredictable gene expression patterns, disruption of endogenous transcription, and malignancy [7]. One approach to improve the safety is to deliver transgenes into predefined genomic loci called genomic safe harbors (GSHs).

An ideal GSH needs to have several properties. First, it should be highly accessible to transgene integration and allow high efficiency in the transgene delivery via homology directed repair (HDR) in the desired cells/tissues. Second, it should be in an actively transcribed region and not be targeted by silencing mechanisms, allowing cell type- and tissue-specific expression. Most importantly, for safety consideration, a GSH should not overlap any known functional sequences in the genome, including exons, promoters, enhancers, transcription units, and ultra-conserved regions, or affect nearby gene expression [4]. So far, only a few human GSHs have been defined, including AAVS1 [8], CCR5 [9], and the human ortholog of the mouse Rosa26 locus [10]. However, none of the current GSH sites show adequate evidence for therapeutic safety. For example, the inserted gene in the AAVS1 site could affect the expression of myosin binding subunit 85 (*PPP1R12C*) and could also be silenced [11]. Similarly, studies regarding the mutation at the CCR5 site also showed increased risk of West Nile virus and Japanese Encephalitis [12, 13]. Thus, stringent GSH selection and evaluation are needed.

With the increasing availability of genomics and epigenomics data, different criteria have been applied to genome-wide searches for GSHs in the human genome [14, 15]. Generally, those criteria require a minimal linear distance from functional DNA elements such as promoters, enhancers, and coding sequences. However, the distance selected is usually arbitrary, and the locus-specific features along the genome are not considered. For example, a locus that is linearly distant from a gene can still be involved in long-range chromatin interaction and contribute to gene activation [16]. Indeed, several studies have shown regulation of genes through long-range interactions [16–18]. In addition, most current methods are based only on genomic features and do not consider tissue-specific gene expression and regulatory elements. A knowledge-based approach that takes the three-dimensional (3D) chromatin organization of the human genome and tissue-specific expression pattern into consideration can overcome these limitations and better define GSHs.

As the starting point of GSH screening, common genetic variants in healthy human populations, particularly large structure variations, can serve as markers for neutral regions. Mobile element insertion is one type of structure variation that is ideal for this purpose. Mobile elements (MEs) are segments of DNA that contribute to at least 50% of the human genome [19, 20]. MEs can move around within the genome and create new insertions. As a result, thousands of polymorphic mobile elements insertions (pMEIs) are present in human populations [21, 22]. pMEIs with high allele frequency (AF) among human populations are considered as common pMEIs. Common pMEIs that are not associated with expression of nearby genes in the tissue of interest can be considered as natural landmarks for genomic loci that can potentially harbor transgene integrations without deleterious effects.

Here, we developed a framework to identify and validate cell type-specific GSHs by integrating pMEI distribution among healthy individuals with gene expression, 3D chromatin organization, and epigenetic modification information. Using data from the 1000 Genomes project and the Genotype-Tissue Expression (GTEx) projects, we identified 19 blood GSH candidate loci. For three candidate loci, we demonstrated the stable expression of transgene without alternating transcription of nearby genes in erythroid cells. We further extended the framework to gene expression data in brain tissues and identified 5 candidate brains GSHs. In addition, we developed a computer program for knowledge-based GSH selection.

## Results

### Overall design of the genomic safe harbor identification procedure

**Fig. 1:**
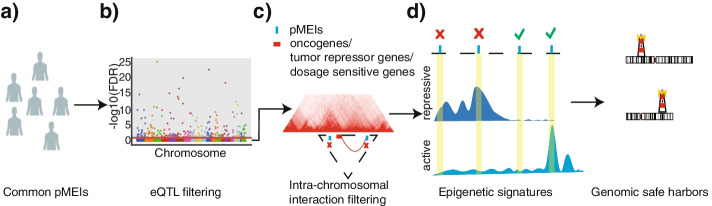
A schematic representation of the overall genomic safe harbor identification strategy. **a** Selection of common pMEIs from healthy individuals with AF > 0.1. **b** Removing pMEIs significantly associated with gene expression (FDR < 0.1 in eQTL mapping). **c** Removing pMEIs showing spatial proximity with oncogenes, tumor suppressor genes, and dosage-sensitive genes based on TADs and chromatin interaction mapping. **d** Removing pMEIs overlapping repressive chromatin regions

Our goal is to identify genomic loci that meet two main criteria for genome engineering: have minimal effects on normal functions of host cells and maintain stable transgene expression. The overall strategy is illustrated in Fig. 1. Because common pMEIs, especially those with high AF in the genomes of healthy people, can harbor large insertions (300 base pairs (bps)—6000 bps) without apparent deleterious effects, we reasoned that these pMEI sites are plausible candidates for GSH selection (Fig. 1a). From common pMEIs, we remove pMEIs associated with tissue-specific gene expression based on expression quantitative trait loci (eQTL) analysis (Fig. 1b). To assess the potential interactions between transgene and the genome of host cells, we use genomic spatial proximity information identified by technologies such as whole-genome Hi-C and promoter capture Hi-C. These technologies can extract genome-wide interactions among different genomic loci. These unbiased long-range interactions allow us to remove pMEI sites that may affect functionally significant genes, such as oncogenes, tumor suppressor genes, and dosage-sensitive genes, through long-range interactions (Fig. 1c). To avoid heterochromatin regions that can potentially decrease the transgene cassette integration and transcription efficiency, genomic regions with repressive and quiescent state markers are also excluded (Fig. 1d). Active chromatin regions have been reported to be associated with high editing efficiency and expression of transgenes. So, we further labelled GSH sites that overlap with active chromatin markers.

### Identification of GSHs in blood cells

To test our framework, we identified common pMEIs in the 1000 Genomes project [23, 24] and conducted eQTL analysis between these common pMEIs and genome-wide expression profiles in matched lymphoblastoid cell lines [25]. We excluded pMEIs that are associated with gene expression within 500 thousand base pairs (kbs) (eQTL FDR < 0.1) (Additional file 1: Figure S1). We then used Hi-C data from GM12878 cells [26] to define topological associated domains (TADs), which are fundamental units of 3D chromatin organization. Because most chromatin interactions happen within the same TAD [26], we removed pMEIs that are within the same TAD as tumor suppressor genes, oncogenes, or dosage-sensitive genes [27–29]. We then removed pMEIs that formed loops with gene promoters within the same TAD using promoter capture Hi-C data [30]. We also removed pMEIs that are within high gene density TADs, defined as TADs with more than the mean gene density of all TADs (28.13 genes/million bps). To avoid the less-frequent inter-TAD interaction, we further removed pMEIs that formed loops with promoters of tumor suppressor genes, oncogenes, or dosage-sensitive genes that are not within the same TAD. To ensure the accessibility of the candidate loci for genome editing, we removed pMEIs that are located within regions with repressive marks, including the heterochromatin regions, regions with polycomb modification signals, and regions labelled as the quiescent state. After filtering, we identified 16 candidate GSHs in blood cells from the 1000 Genomes project data (Table 1, Additional file 1: Figure S2a).

**Table 1:** Identified GSH sites in blood from the 1000 Genomes project and the GTEx data

GSH_ID	Position	FDR	AF	TAD gene density	Active regions	Gene	Location	Dataset
BLD_GSH_1	chr1:150200138-150200139	0.84	0.14	NA	4_Tx,5_TxWk	ANP32E	Intron	1KG
BLD_GSH_2	chr1:198243300-198243301	1	0.11	4.94	5_TxWk	NEK7	Intron	1KG, GTEx
BLD_GSH_3	chr11:129759556-129759557	1	0.25	NA	4_Tx,5_TxWk	NFRKB	Intron	1KG
BLD_GSH_4	chr12:122722288-122722289	1	0.23	NA	4_Tx,5_TxWk	VPS33A	Intron	1KG
BLD_GSH_5	chr13:111559414-111559652	0.87	0.2	NA	4_Tx,5_TxWk	ANKRD10	Intron	1KG
BLD_GSH_6	chr15:49609604-49609605	1	0.17	10.7	5_TxWk	GALK2	Intron	1KG, GTEx
BLD_GSH_7	chr15:59169388-59169389	0.47	0.16	NA	4_Tx,5_TxWk	-	Intergenic	1KG
BLD_GSH_8	chr2:39071477-39071819	0.16	0.32	NA	4_Tx,5_TxWk	DHX57	Intron	1KG
BLD_GSH_9	chr2:223481690-223481979	0.93	0.28	NA	4_Tx,5_TxWk	FARSB	Intron	1KG
BLD_GSH_10	chr3:37361602-37361603	1	0.1	25	4_Tx,5_TxWk	GOLGA4	Intron	1KG, GTEx
BLD_GSH_11	chr3:45542662-45542663	0.13	0.36	NA	4_Tx,5_TxWk	LARS2	Intron	1KG, GTEx
BLD_GSH_12	chr3:45768351-45768676	0.26	0.38	NA	4_Tx,5_TxWk	SACM1L	Intron	1KG
BLD_GSH_13	chr4:88032137-88032469	0.76	0.58	NA	4_Tx,5_TxWk	AFF1	Intron	1KG, GTEx
BLD_GSH_14	chr6:157397700-157397701	1	0.13	NA	2_TssAFlnk,5_TxWk	ARID1B	Intron	1KG, GTEx
BLD_GSH_15	chr8:120800792-120800793	0.89	0.21	NA	4_Tx,5_TxWk	TAF2	Intron	1KG
BLD_GSH_16	chr9:100675550-100675551	n.s.	0.16	NA	4_Tx,5_TxWk	TRMO	Intron	GTEx
BLD_GSH_17	chr9:115937084-115937379	0.34	0.35	NA	4_Tx,5_TxWk	FKBP15	Intron	1KG
BLD_GSH_18	chr1:1654012-1654013	n.s.	0.15	NA	5_TxWk	CDK11A	Intron	GTEx
BLD_GSH_19	chr15:79167169-79167170	n.s.	0.12	NA	1_TssA,2_TssAFlnk	MORF4L1	Intron	GTEx

*GSH_ID:* Unique GSH ID, *Position:* genomic coordinates for the GSH (hg19), *FDR:* eQTL FDR value, *n.s.* non-significant, *AF:* pMEI allele frequency, *TAD gene density:* Gene density of GSH TAD, NA GSH not assigned to a TAD, *Active regions:* active transcription region based on ChromHMM states, *Gene:* GSH overlapping gene, *Location:* position of GSH (intron, exon, intergenic), *Dataset:* data source, *1KG* the 1000 Genomes Project, *GTEX* the Genotype-Tissue Expression Project

Next, we examined the contribution of different genomics features to the GSH filtering (Additional file 1: Figure S1). Repressing marks was the most important factor, with 94.7% of pMEI loci overlapping repressive regions. Another major factor is AF, with 55.3% of pMEIs’ AF outside of our requirement (10% < AF < 90%). This is consistent with the hypothesis that the majority of pMEIs are deleterious and under purifying selection. About 32% of pMEIs were within a TAD harboring oncogenes, tumor suppressor genes, or dosage-sensitive genes. In addition, 27.6% of pMEIs form a loop with promoters of those genes within the same TAD (16.3%) or across different TADs (11.3%).

**Fig. 2:**
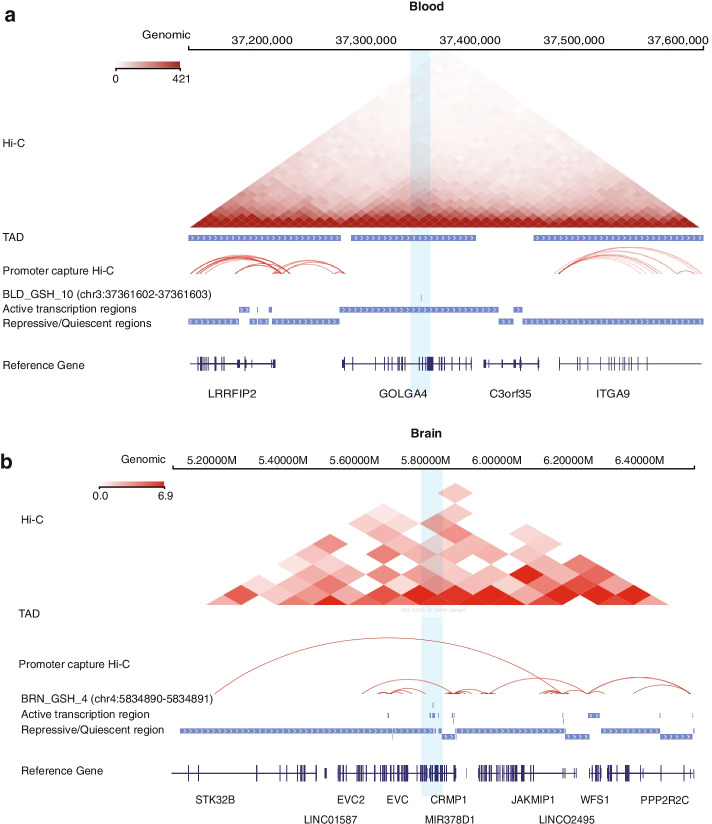
Epigenetic and chromatin interactions near the candidate GSH sites in blood and brain. **a** Genome browser screenshot of a representative GSH in blood. From top to bottom: Interaction heatmap and TADs from Hi-C in GM12878 cells. Chromatin interaction loops from promoter capture Hi-C in blood cells (see Method section for details). The coordinate of the GSH. Active and repressive genomic regions defined by 15-state ChromHMM from blood cells in the Roadmap project (Additional file 9: Table S8), and reference genes. **b** Genome browser screenshot of a representative GSH in brain. From top to bottom: Interaction heatmap and TADs from Hi-C in brain hippocampus. Chromatin interaction loops from promoter capture Hi-C in brain cells (dorsolateral prefrontal cortex, hippocampus, and neural progenitor cells). The coordinate of the GSH. Active and repressive genomic regions defined by 15-state ChromHMM from brain cells in the Roadmap project (Additional file 9: Table S8), and reference genes. Regions surrounding the GSH sites are highlighted with blue shade

Fifteen of sixteen candidate GSHs are in intronic regions, and one is in an intergenic region. All GSHs are in active chromatin regions, and 13 are located outside of TADs identified in GM12878 cells. For example, BLD_GSH_10 (chr3:37361602-37361603) is in the intron of *GOLGA4* (Fig. 2a, Additional file 1: Figure S2a), which is the only gene within the TAD. This pMEI has a 10.4% AF, is in active chromatin regions, and does not form any loops with surrounding genes or their promoters.

To test the reproducibility of our framework, we conducted a similar analysis using a published pMEI-associated eQTL dataset generated in blood cells from the GTEx project [21]. In the GTEx dataset, our framework identified nine candidate blood GSHs. Six of these sites (66.7%) overlap (defined as within 15 bps) with GSHs identified in the 1000 Genomes data (Table 1). For the three unique GSHs in the GTEx data, two (BLD_GSH_19, BLD_GSH_16) were removed from the 1000 Genomes project data by the AF filter and eQTL filter respectively, and for the other (BLD_GSH_18) the pMEI was present only in the GTEx data. The highly consistent results between two independent datasets further confirmed the robust performance of our framework.

### Identification of GSH sites in brain

To test the selection criteria in a different tissue, we mapped GSHs in brain cells using GTEx pMEI and brain-specific gene expression data [21], epigenetic profiles, and 3D chromatin organization profiles [31, 32]. Altogether, we identified five candidate GSH sites (Additional file 1: Figure S2b, Table 2, one example (BRN_GSH_4) is shown in Fig. 2b). Similar to GSHs identified in blood cells, all the brain-GSHs were in intronic regions and labelled as active chromatin region. GSHs identified in brain and blood cells were unique to each other, highlighting the importance of tissue-specific mapping for GSHs.

**Table 2:** Identified GSH sites in brain from the GTEx data

GSH_ID	Position	FDR	AF	TAD gene density	Active regions	Gene	Location	Dataset
BRN_GSH_1	chr1:247027889-247027890	n.s	0.46	NA	4_Tx,5_TxWk	AHCTF1	Intron	GTEx
BRN_GSH_2	chr12:987960-987961	n.s	0.14	NA	4_Tx,5_TxWk	WNK1	Intron	GTEx
BRN_GSH_3	chr22:18283915-18283916	n.s	0.32	NA	5_TxWk	MICAL3	Intron	GTEx
BRN_GSH_4	chr4:5834890-5834891	n.s	0.22	NA	4_Tx,5_TxWk	CRMP1	Intron	GTEx
BRN_GSH_5	chr7:5553845-5553846	n.s	0.15	NA	1_TssA,2_TssAFlnk	LOC221946	Intron	GTEx

*GSH_ID:* Unique GSH ID, *Position:* Genomic coordinates for the GSH (hg19), *FDR:* eQTL FDR value, *n.s.* non-significant, *AF:* pMEI allele frequency, *TAD gene density:* gene density of GSH TAD, NA GSH not assigned to a TAD, *Active regions:* active transcription region based on ChromHMM states, *Gene:* GSH overlapping gene, *Location:* position of GSH (intron, exon, intergenic), *Dataset:* data source, *GTEX* the Genotype-Tissue Expression Project

### Validation of GSH site in HUDEP2 cells

To experimentally assess the candidate blood GSHs identified by our framework, we integrated a green fluorescent protein (GFP) expression cassette into candidate GSH loci through homologous recombination in HUDEP2 cells. HUDEP2 is an erythroid progenitor cell line and can be differentiated into mature erythroid cells. We tested three GSHs identified in blood cells. As controls, we included two randomly selected non-GSH pMEIs, one GSH identified in brain tissue but is located within heterochromatin in blood cells, and the AAVS1 locus. We designed two CRISPR guide RNAs for each locus and chose the one with higher editing efficiency for the cassette integration (editing efficiency for all gRNAs are greater than 50%, Additional file 2: Table S1). GFP cassettes were integrated into the GSH loci through HDR-mediated insertion. We sorted GFP-positive cells and established the stable cell line for each locus. We further carried out PCR assays to validate each integration site (Additional file 1: Figure S3).

**Fig. 3:**
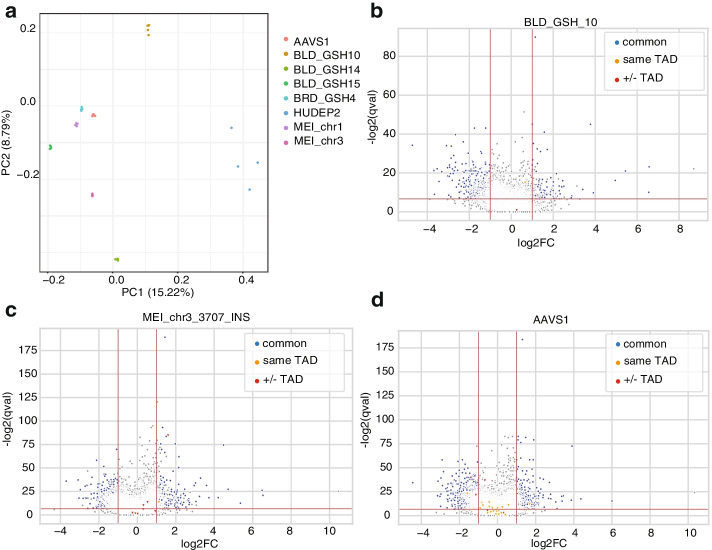
Experimental validation of GSHs in HUDEP2 cells. **a** PCA plot showing the RNA-seq data for all tested cell lines. **b–d** Volcano plots showing differential expressed genes (DEGs) in a blood GSH (BLD_GSH_10), non-GSH MEI (MEI_chr3_3707_INS) and AAVS1. Common: DEGs share by more than two cell lines. Same TAD: genes within the same TAD of the GFP integration site; +/− TAD: genes in the TADs flanking to the GFP integration site

To compare the expression profiles between GFP integrated cell lines and wild-type (WT) HUDEP2 cells, we performed RNA-seq assays with 4 replicates per cell line (Fig. 3a). In general, genome-wide expression profiles among all samples are highly correlated (minimal spearman correlation coefficient *R*=0.89, Additional file 1: Figure S4). We then performed differential gene expression analysis (See “Methods”). On average, there are ~ 250 genes upregulated and ~ 800 genes downregulated in GFP integrated cell lines (FDR<0.01, Log2 Fold Change (LFC) >1 or LFC<−1, Additional file 3: Table S2). Interestingly, most (~80%) of these differential expressed genes are shared among at least three cell lines with different integration sites (Fig. 3b–d, Additional file 1: Figure S5). Gene ontology (GO) enrichment analysis showed that genes which involved protein degradation such as Ubl conjugation are highly enriched (FDR 1.1E-8), indicating that the expression level changes of those genes are likely triggered by cellular response to GFP [33, 34]. To assess the *cis *effect of GFP integration at each GSH, we focused on genes within the same TAD of integration sites. Among the three blood GSHs, there are no significantly changed genes (Fig. 3b, Additional file 4: Table S3) within same TAD. In contrast, the GFP cassette integrated into one randomly selected MEI (MEI_chr3_3707_INS) leads to significantly increased (LFC =1.12, FDR=9.39E−05) expression of *PTX3* (Fig. 3c). GFP integrated in the AAVS1 locus also alternated the expression of two genes, *TNNI3* (LFC=1.684, FDR=3.1E−04) and *PPP6R1* (LFC=−1.59, FDR=1.34E−07) (Fig. 3d, Additional file 4: Table S3). This result is consistent with previous studies showing that cassette integration at the AAVS1 site could affect the nearby gene expression [11].

Expression cassettes integrated in intron regions can potentially affect RNA splicing. To assess this risk, we performed alternative splicing analysis on genes with GFP cassettes integrated in their introns. No significant alternative splicing events were detected in any locus (rMATS FDR<0.01, Additional file 5: Table S4). We further flipped the orientation of the GFP cassette at the BLD_GSH_10 locus and found that neither direction affects the splicing of *GOLGA4* (Additional file 5: Table S4). In addition, the expression levels of *GOLGA4* and other genes within the same TAD did not change significantly (FDR< 0.01, LFC>1 or LFC <−1) between the two cassette integration directions (Additional file 4: Table S3).

**Fig. 4:**
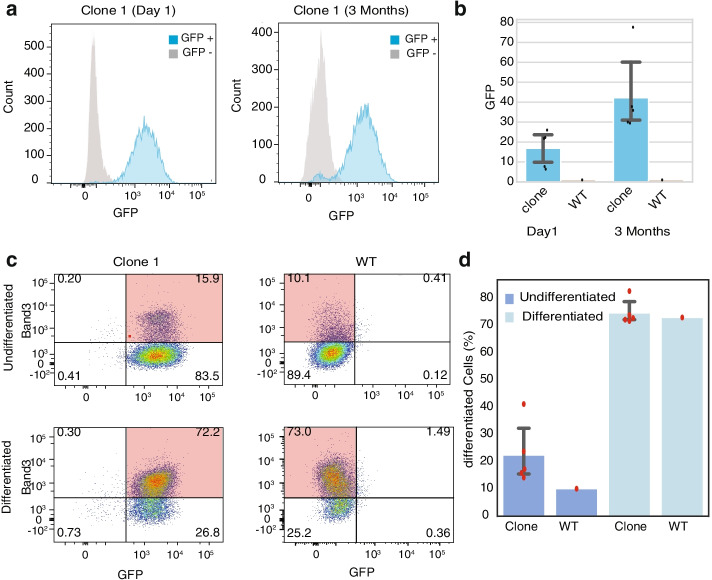
Long-term validation of BLD_GSH_10 clones. **a** Representative distribution of GFP fluorescence signals in HUDEP2 WT cells (gray) and cells from a HUDEP2 clone with a GFP reporter transgene integrated in the GSH site (blue) in day 1 and day 90, respectively. **b** Bar plots showing the normalized GFP fluorescence signals of five independent clones and WT HUDEP2 cells. **c** Representative immuno-flow cytometry results showing cell differentiation comparison between WT cells and cells from one GFP clone. *Y*-axis is the signal for red blood cell maturation marker Band3. *X*-axis is the signals for GFP. The mature red blood cell compartment is highlighted in red. **d** Bar plots showing the percent of Band3 high cell populations before and after differentiation for five GFP clones and WT cells

To assess the stability of GFP expression at the GSH sites, we continually cultured the cells for 1 month. Among the three cell lines with GFP integrated into the heterochromatin regions, two cell lines lost more than 30% GFP-positive cells. In contrast, at least 85% cells with GFP integrated into the active regions remain GFP positive (Additional file 6: Table S5, Additional file 1: Figure S6). We further generated 5 clones of *GOLGA4* locus and cultured the cells for 3 months. In all 5 clones, strong GFP signals were well-maintained (average normalized geometric mean of 42.1) for 3 months of continued culture (Fig. 4a, b, Additional file 1: Figure S7, Additional file 7: Table S6), suggesting that the integrated transgene can maintain stable transcription in the target cells. To assess whether the integrated transgene affects normal red blood cell functions, we induced terminal maturation for 5 days and fractionated cells according to expression of the late-stage erythroid marker Band3. All five clones show similar expression level of Band3 (14.2–41.2%) compared to WT (10.1%) in undifferentiated cells and in differentiated cells (71.7–82.7% compared to 73% in WT) (Fig. 4c, d and Additional file 1: Figure S8).

### User-friendly pipeline for identifying GSH sites in different tissues

To extend the application of our framework, we developed a user-friendly program: Genomics and Epigenetic Guided Safe Harbor mapper (GEG-SH mapper, https://github.com/dewshr/GEG-SH). To use the program, a user first provides a list of genomic variants with genomic coordinates and optional information, such as AF and eQTL significance. Then, GEG-SH mapper will select candidate GSH sites by integrating TAD information; chromatin interaction information; epigenetic information such as repressive chromatin region and active chromatin region; and annotation of oncogenes, tumor suppressor genes, and dosage-sensitive genes. Because epigenetics features can be tissue-specific, a user can also replace the default data sets with those from the tissue/cell type of interest. The program reports candidate GSHs and the link to the UCSC genome browser for visualization of the candidate GSHs. A file containing annotations for all input variants is also generated to allow users to conduct custom query and filtering (Additional file 8: Table S7).

## Discussion

Genome engineering technologies have developed rapidly over the last decade. Gene and cellar therapies have the potential to treat once-incurable diseases [1, 2, 35]. However, the functions of the human genome are not fully understood. Avoiding unintended changes in important genomic regions remains a major consideration during genome engineering. A large amount of effort has been spent to establish complicated experiment systems to identify and prevent these potential deleterious effects [36–38]. However, since the functional consequences of genomic alternation may only be detected in a specific cell type during a specific development stage and/or under specific conditions, it is challenging to include all these factors in the experimental design.

In this study, we developed a novel GSH discovery approach. First, we select GSH candidate regions based on common pMEIs in healthy human populations. Because these pMEIs have been subjected to hundreds of thousands of years of purifying selection and remained common in human populations, they marked genomic regions that are selectively neutral with little or no impact on genomic functions. Among common pMEIs, we then excluded loci that are associated with tissue-specific expression of nearby genes to further increase the likelihood of selecting region with no functional impacts. Second, unlike most current GSH mapping approaches that mask genome with arbitrary defined linear windows near important DNA elements [14, 15], our approach is knowledge-based and considers 3D chromatin organizations of the genome and the 3D spatial distance between genomic loci. Third, stable expression of the transgene is essential for an effective gene therapy. Thus, it is crucial that the GSHs are outside of the repressive/heterochromatin regions. To this end, we use tissue-specific epigenetic signatures to identify genomic regions that are open for transcription in the tissue of interest. This step is crucial for GSH selection, as we found that 94.7% (5880/6206) of the pMEIs from the 1000 Genomes project overlaps repressive chromatin marks. More importantly, we identified no shared GSHs between blood and brain. The large amount of tissue-specific repressive regions in the genome and the tissue-specific nature of the candidate GSHs highlight the importance of including tissue-specific epigenetic information for GSH identification. Using a cell-culture system, we showed that GFP transgene cassette can be effectively integrated into candidate GSH loci. After integration, the cassettes were stably expressed for several months, and they did not alter the expression of nearby genes in host cells. These results demonstrate that our method can identify tissue-specific GSH candidates. It is worth mentioning that the commonly used AAVS1 locus is located within a high gene density TAD. In our cell line-based validation system, GFP cassette integration in AAVS1 significantly altered the expression of two nearby genes. Thus, there is an urgent need to identify more and better GSHs.

Importantly, our goal is to provide a framework for GSH identification. Although our validation experiments demonstrate promising results, our experiments have several limitations and the GSHs we tested should not be considered fully validated. One safety concern of gene therapy is that the integration of gene expression cassettes can potentially change RNA splicing of host genes [39]. Even though no significant alternative splicing events were detected in our study, we cannot completely exclude the possibility for other expression cassettes, especially those with splicing acceptor consensus sequences, could affect the nearby gene splicing. Thus, carefully assessing the splicing events is important for new expression cassettes. Another concern is the transcriptional leakage of gene expression cassettes [40]. In our validation experiment, we observed transcriptional leakage that can extend up to 600 bps downstream of GFP cassettes. One potential solution is adding insulator elements to the cassettes [41]. Another limit in our pipeline is that some functional genomic data used are from cell lines instead of more clinically relevant primary cells such as CD34+ hematopoietic stem and progenitor cells (HSPCs). This is largely due to the data availability. Our pipeline does have an option to let users provide their own functional genomic data and identify best GSHs for their own systems. Last but not least, we used a GFP expression cassette in our experimental validation. Since transgene-genome interaction can be transgene-dependent, expression cassettes with different transgene can potentially induce different local epigenetic and chromatin interaction changes. Thus, researchers should perform their own tests to select the GSHs that work the best for their specific studies. Nevertheless, the evidence that more than 10% of the healthy human population has large DNA fragments integrated in these candidate GSHs for thousands of years provides extra information that is not available from any cell or animal models.

## Conclusion

We developed a knowledge-based computational tool (GEG-SH mapper) for selecting tissue-specific GSH sites for gene therapy and genomic engineering studies and demonstrated its utility in blood and brain. In total, we identified 19 GSH in blood and 5 in brain tissues. We also validated three GSH sites and showed high gene expression correlations in cells with and without the transgene integration as well as similar proliferation and differentiation state in these cells. Combining with targeted cassette integration technology, our approach will allow more efficient development of genomic engineering studies and gene therapies in the near future.

## Methods

### pMEI-associated eQTL identification in the 1000 Genomes project

Genotypes for pMEI loci in 2504 individuals were extracted from the 1000 Genomes project phase 3 release of structure variation (ftp://ftp.1000genomes.ebi.ac.uk/vol1/ftp/phase3/integrated_sv_map/) [23, 24]. In this dataset, 16,631 pMEIs were present in certain samples but not in the reference genome (referred as insertions), and 1304 were present in the reference genome but missing in certain samples (referred as deletions). RNA-Seq data from 462 individuals were downloaded from GEUVADIS RNA sequencing project for the 1000 Genomes project samples [25]. Among the 462 individuals, 445 individuals matched the pMEI genotype file. The following analyses were based on these 445 individuals.

For the eQTL analysis, pMEI were filtered to include pMEI with >1% and <99% AF in the 445 individuals. The gene expression level was calculated as the Reads Per Kilobase of transcript, per Million mapped reads (RPKM) values using cufflinks software [42]. Both protein-coding and non-coding genes defined in the GENCODE annotation [43] were used. Matrix-eQTL [44] was used to perform eQTL tests for the association between pMEIs and expression changes in *cis* (i.e., a pMEI and a gene are located within 500 kbs of each other) by using an additive linear regression model. Population and gender information were considered in the matrix-eQTL analysis as covariates. pMEIs that are more than 500 kbs away from genes are also included in the downstream analysis.

### GSH-mapper filtration of pMEIs

The eQTL data from the 1000 Genomes project (as described above) and the GTEx project [21] were processed to generate the GSH-mapper input format containing ID, position, eQTL FDR, and AF. Both eQTL datasets included pMEI’s association with the expression of protein coding and non-coding genes. The position column was used to generate a bed file with the chromosome coordinates, which was used for further filtration steps.

#### Oncogenes, tumor suppressor genes, and dosage-sensitive genes

The lists of oncogenes and tumor suppressor genes were downloaded from [27, 28], respectively. The list of dosage-sensitive genes was downloaded from the Exome Aggregation Consortium [29], supplementary table 13 with a pLI (probability loss of function intolerant) value greater than 0.9. The gene coordinate information was assigned by using BioMart Ensemble genes 104 database for GRCh37.

#### TAD

TAD information for blood was obtained from GSE63525 [26], and brain data were obtained from GSE86189 [32]. For blood pre-processed arrowhead [45], data for GM12878 cells were used. For brain hippocampus, raw data (SRA: SRR4094699) was downloaded and processed locally using Hic-pro [46] (Version 2.11.1) with default parameters. A bin size of 100 kbs was used to generate Iterative Correction and Eigenvector decomposition (ICE) normalized contact maps. Normalized contact maps were converted into “h5” format by using hicConvertFormat, and TADs were identified using hicFindTADs. Both tools are parts of HiCExplorer (version 3.6) [47–49] and were run using default parameters through the command line version. TAD information was assigned to pMEIs using Bedtools intersect (Version 2.29.2) [50]. The gene coordinate information was downloaded from BioMart Ensemble genes 104 database for GRCh37. Gene density for each TAD was calculated as:

$$\mathrm{Gene}\ \mathrm{density}=\left(\mathrm{number}\ \mathrm{of}\ \mathrm{genes}\ \mathrm{in}\ \mathrm{TAD}/\mathrm{length}\ \mathrm{of}\ \mathrm{TAD}\right)\times 1000000.$$

Mean gene density was calculated based on gene densities of all TADs in the genome for the given cell/tissue. pMEIs within TADs with gene density greater than the mean gene density were removed. The mean gene density value will vary depending on the input TAD regions.

#### Promoter interaction

Gene promotor chromatin interaction data for blood (PCHiC_peak_matrix_cutoff5.txt.gz) [51] were downloaded from https://osf.io/u8tzp/ and all the interactions files were combined except for endothelial precursors and fetal thymus cells. Similarly, for brain data, supplementary Tables 3 and 4 were downloaded from Jung et al [32], and interactions involving dorsolateral prefrontal cortex, hippocampus, and neural progenitor cells were combined. For promoter-promoter interaction data in supplementary Table 4, where only gene name was provided, promoter regions were defined as regions 2 kbs upstream and downstream of the gene transcription start site (TSS). pMEIs interacting with gene promoters within the same TAD were removed. pMEIs with chromatin interaction to promoters of dose-sensitive genes, tumor suppressor genes, or oncogenes were removed.

#### Chromatin regions

The pMEIs were further filtered and annotated based on chromatin regions. Chromatin region information inferred by ChromHMM [52, 53] for both blood and brain were obtained from the Roadmap Epigenome Consortium (Additional file 9: Table S8) [31]. Heterochromatin, Repressed Polycomb, Weak Repressed Polycomb, and Quiescent regions defined by ChromHMM were considered as repressive regions. Active TSS, Flanking TSS, Strong transcription, and Weak transcription regions were considered as active regions.

### HUDEP2 clone generation with GFP marker at GSH

For Clustered Regularly Interspaced Short Palindromic Repeats (CRISPR) integration, 1 μl of 50 μM sgRNA and 0.5μl of 40μM 3xNLS-Cas9 protein were mixed and incubated at room temperature for 10 min. The gRNA and Cas9 (RNP) mixture were then transferred to ice. A total of 200,000 HUDEP2 cells were resuspended in 10 μl buffer R (Invitrogen: MPK1096R). Then 1.5 μl of RNP mixture was added along with 1 μl of 1 μg/μl Donor EGFP plasmid (Additional file 1: Figure S9), which contains homologous arms and GFP expression cassette. The cells were subjected to electroporation using Invitrogen Neon transfection system at 1200v, 40 ms, 1 pulse. After electroporation, cells were transferred into 2-well plates, with 1 mL/well containing 10% FBS without any antibiotics. After 1 week of cell culture, GFP+ single cell was sorted into 96-well u-bottom plates. When cell pellets are visible (around 10–14 days), the subclones were then transferred into 12-well plates for clonal expansion. GFP insertions in cells were validated by PCR to amplify GFP cassette. The sequences of gRNAs and PCR primers are listed in Additional file 2: Table S1.

### HUDEP2 cell differentiation and FACS staining

For cell differentiation analysis, HUDEP2 cells were cultured in IMDM medium containing 2% human AB plasma, 3% human AB serum, 1% penicillin/streptomycin, 3 U/mL heparin, 10 μg/mL insulin, 3 U/mL EPO, 1 mg/mL transferrin, 50 ng/mL hSCF, and 1 μg/mL doxycycline for 3 days. The cell density was maintained between 0.7×10^6^/mL and 1.4×10^6^/mL. After day 4, hSCF was withdrawn from the culture medium and cell density was maintained between 1×10^6^/mL and 2×10^6^/mL. For cell sorting, 0.5×10^6^/mL cells were resuspended in 1000 mL of PBS with 2% FBS. Then, 2 mL of Band3-APC (Dr. Xiuli An from Laboratory of Membrane Biology, New York Blood Center provided the antibody) was added, and the cells were kept on ice for 20 min. The cells were then washed twice with 200 mL of PBS containing 2% FBS and resuspended in 200 mL of PBS containing 2% FBS for Fluorescence Activated Cell Sorting (FACS).

### RNA sequencing and analysis

RNA sequencing was performed as previously described [54]. Briefly, quick-RNA MiniPrep kit (Zymo Research, R1054) was used to extract RNA from one million normal HUDEP2 cells or HUDEP2 GFP cells. For each cell line, RNAs were prepared from four batches of bulk sorted GFP cells as biological replicates. The TruSeq Stranded RNA Library Prep Kit (Illumina) was used to create libraries for sequencing. Sequencing was performed using NovaSeq 6000 (Illumina) with 100PE format.

Kallisto quant (0.43.1) [55] with the default setting using bootstrap-samples set to 100, and Ensembl gene annotation (version 75) for the human reference genome (hg19) was used to get the transcript abundance data. Differential gene expression analysis was performed using Sleuth [56] with the default parameters. FDR<0.01, LFC>1, or LFC<-1 were used to identify significantly changed genes. In addition, the normalized TPM counts generated by Sleuth were used for correlation analysis among the GFP inserted cell lines and HUDEP2 WT.

For alternative splicing analysis, RNA sequencing data were mapped using STAR (version 2.5.3a) [57] and alternative splicing events were analyzed using rMATS (4.0.2) [58] using default settings, where each GFP inserted cells were compared with WT HUDEP2 cells. GENCODE v39lift37 is used as annotation file. Five different events (skipped exon, mutually exclusive exon, alternative 3′ splice site, alternative 5′ splice site, and retained intron) were evaluated to identify significant alternative splicing events.

## Supplementary Information

Additional file 1: Supplementary figures: Figure S1. Pipeline workflow for identification of GSH sites. Figure S2: Circos plot representation of GSH sites identified in blood (a) and brain (b) data. Figure S3: PCR validation of GFP inserted cell line. Figure S4: Genome wide gene expression Correlation between GFP integrated cell lines and HUDEP2 cells. Figure S5: Volcano plot representation of Differentially Expressed Genes for four GSH integration sites. Figure S6: GFP maintenance after one month based on different integration sites. Figure S7: GFP expression in Day 1 and Day 90 for 5 GFP clones of BLD_GSH_10. Figure S8: Flow cytometry of GFP and Band3 staining of HUDEP2 GFP clones and HUDEP2 WT cells on day 0 and day 4 of differentiation. Figure S9: Plasmid vector used for the GFP insertion in HUDEP2 cells.

Additional file 2: Table S1. gRNA editing efficiency for different loci and primers used for validation of GFP integration

Additional file 3: Table S2. Differentially expressed genes in GFP integrated cell lines.

Additional file 4: Table S3. Expression level changes of genes near integration sites

Additional file 5: Table S4. Alternative splicing events detected by rMATS.

Additional file 6: Table S5. Loss of GFP level based on integration sites.

Additional file 7: Table S6. GFP flow cytometry data of day 1 and day 90 for WT and GFP inserted HUDEP2 clones.

Additional file 8: Table S7. Detailed pMEI annotations for GSH selection.

Additional file 9: Table S8. Data used to define repressive and active chromatin regions.

Additional file 10. Review history.

## Data Availability

RNA sequencing data are available on GEO database (GSE183935) [59] and code and other data used to identify candidate GSH sites are available at https://github.com/dewshr/GEG-SH/tree/v1 [60] as well as in Zenodo (10.5281/zenodo.7041570) [61]. The source code is released under an open source MIT License.
