# Supplementary material for: Genomics and epigenetics guided identification of tissue-specific genomic safe harbors
Source: Genome Biol. 2022 Sep 21;23:199. doi: 10.1186/s13059-022-02770-3 (PMC9490961; doi:10.1186/s13059-022-02770-3)
Supplement: Supplementary file 1 — Additional file 1: Supplementary figures: Figure S1. Pipeline workflow for identification of GSH sites. Figure S2: Circos plot representation of GSH sites identified in blood (a) and brain (b) data. Figure S3: PCR validation of GFP inserted cell line. Figure S4: Genome wide gene expression Correlation between GFP integrated cell lines and HUDEP2 cells. Figure S5: Volcano plot representation of Differentially Expressed Genes for four GSH integration sites. Figure S6: GFP maintenance after one month based on different integration sites. Figure S7: GFP expression in Day 1 and Day 90 for 5 GFP clones of BLD_GSH_10. Figure S8: Flow cytometry of GFP and Band3 staining of HUDEP2 GFP clones and HUDEP2 WT cells on day 0 and day 4 of differentiation. Figure S9: Plasmid vector used for the GFP insertion in HUDEP2 cells. [file 13059_2022_2770_MOESM1_ESM.pdf]

Genomics and epigenetics guided identification of tissue-specific genomic safe harbors

Dewan Shrestha1,2#, Aishee Bag3#, Ruiqiong Wu2, Yeting Zhang3, Xing Tang2, Qian Qi2, Jinchuan Xing3,4*, Yong Cheng2,5*

1. Department of Genetics, Genomics, and Informatics, College of Graduate Health Sciences, The University of Tennessee Health Science Center, Memphis, TN, USA
2. Department of Hematology, St. Jude Children's Research Hospital, Memphis, TN, USA
3. Department of Genetics, Rutgers, The State University of New Jersey, Piscataway, NJ, USA
4. Human Genetic Institute of New Jersey, Rutgers, the State University of New Jersey, Piscataway, NJ, USA
5. Department of Computational Biology, St. Jude Children's Research Hospital, Memphis, TN, USA

# These authors contributed equally to the project.
* Corresponding authors: J.X. and Y.C.

Email addresses:

DS: dshrest2@uthsc.edu; AB: ab1603@rwjms.rutgers.edu; YZ: leafiezyt@gmail.com; RW: Ruiqiong.Wu@STJUDE.ORG; XT: tangx1986@gmail.com; QQ: Qian.Qi@STJUDE.ORG; JX: jinchuan.xing@rutgers.edu; YC: Yong.Cheng@STJUDE.ORG

Additional file 1:

Supplementary figures S1-9

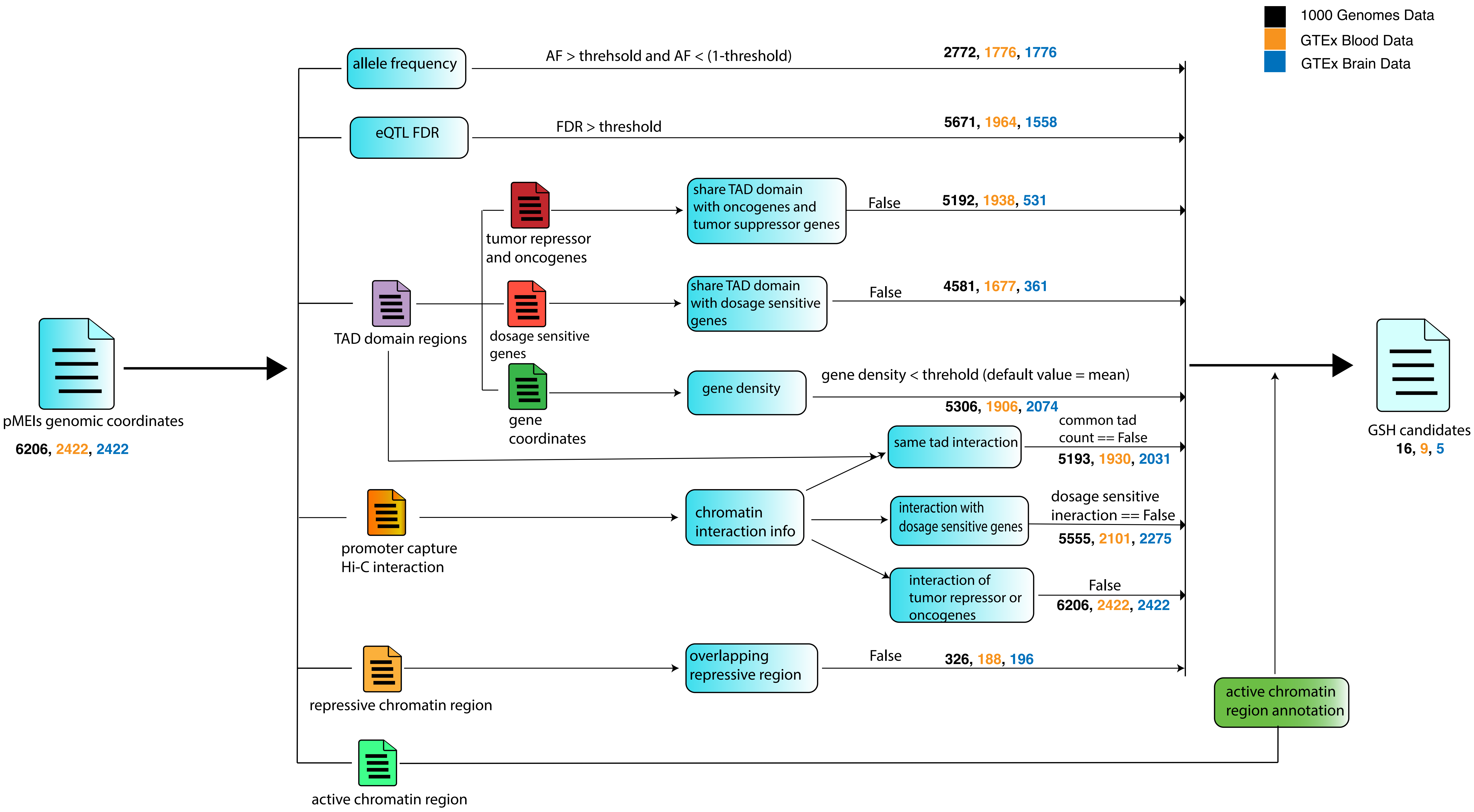

Figure S1: Pipeline workflow for identification of GSH sites.
The number in each of the filtration step is the number of pMEIs that passed the filtration criteria. Each color represents the specific dataset used. Black: 1000 Genomes Project; Orange: GTEx blood; Blue: GTEx brain. Because one pMEI can be filtered out at multiple steps, the numbers represented on each filtration steps are not exclusive.

a

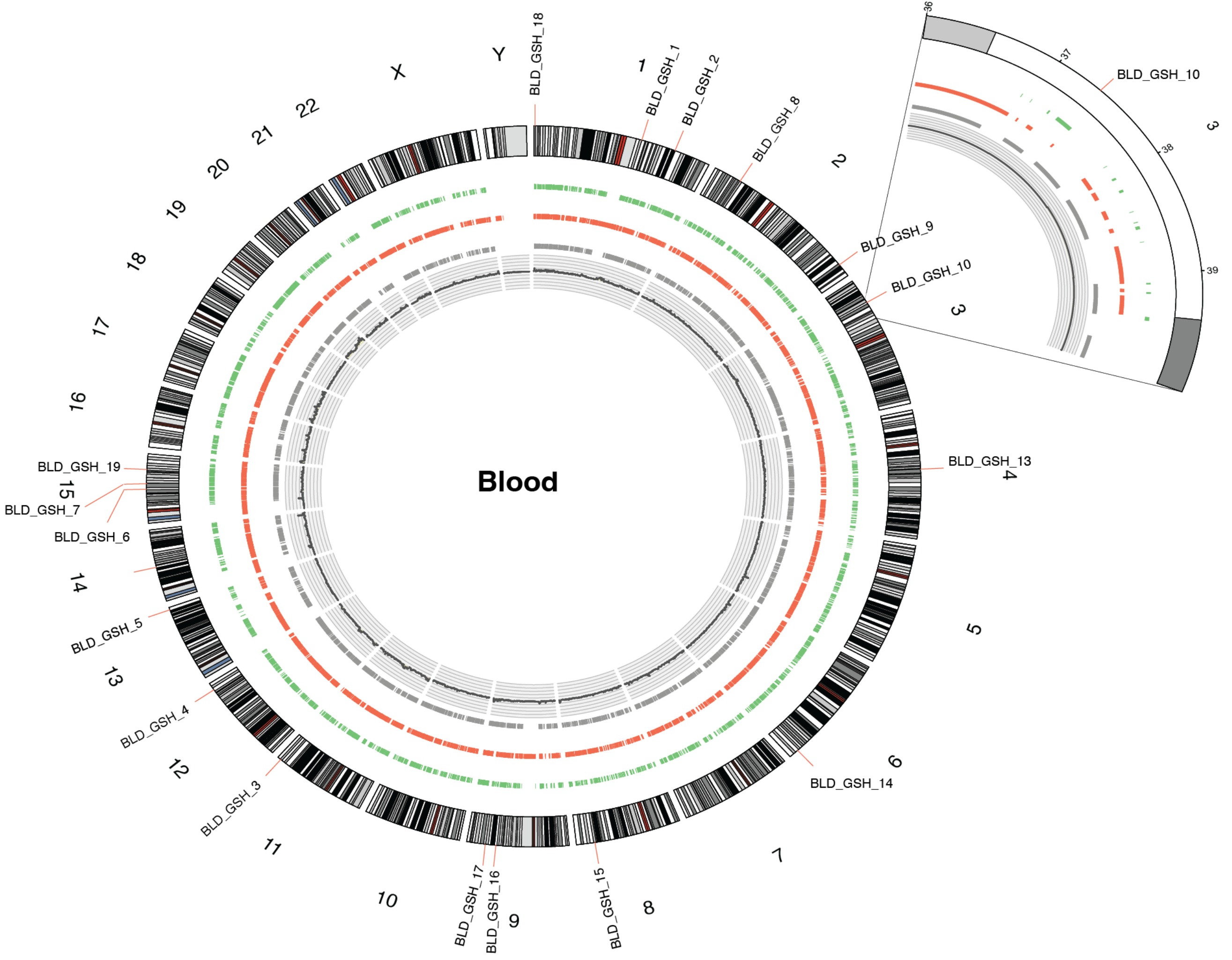

Blood
BLD_GSH_18
BLD_GSH_1
BLD_GSH_2
BLD_GSH_8
BLD_GSH_9
BLD_GSH_10
BLD_GSH_13
BLD_GSH_14
BLD_GSH_15
BLD_GSH_16
BLD_GSH_17
BLD_GSH_3
BLD_GSH_4
BLD_GSH_5
BLD_GSH_6
BLD_GSH_7
BLD_GSH_19

b

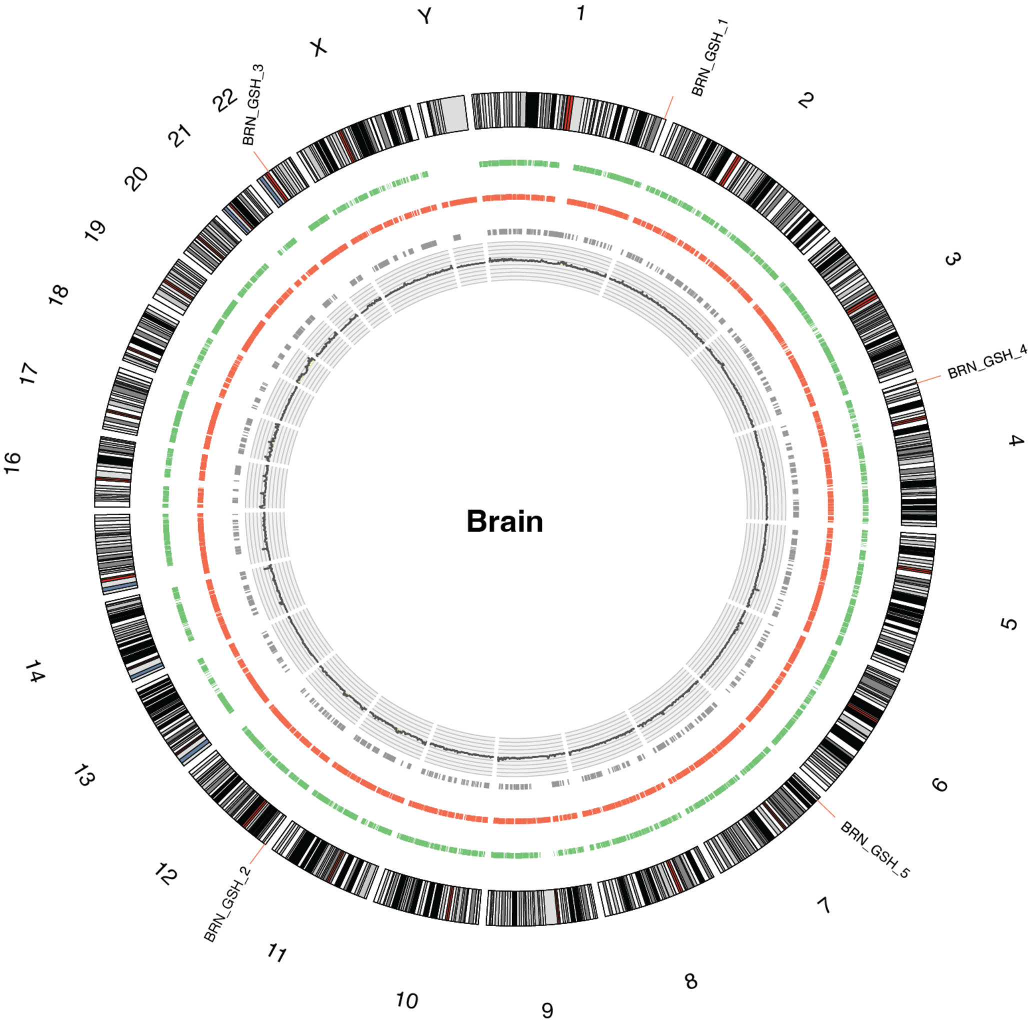

Brain
BRN_GSH_1
BRN_GSH_2
BRN_GSH_3
BRN_GSH_4
BRN_GSH_5

Figure S2: Circos plot representation of GSH sites identified in blood (a) and brain (b) data. The inner circle represents gene density. The grey bands outside of inner circle represents TADs. Repressive chromatin regions are represented by red bands and active regions by green bands. Validated BLD_GSH_10 locus on chromosome 3 in the blood data was enlarged as an example.

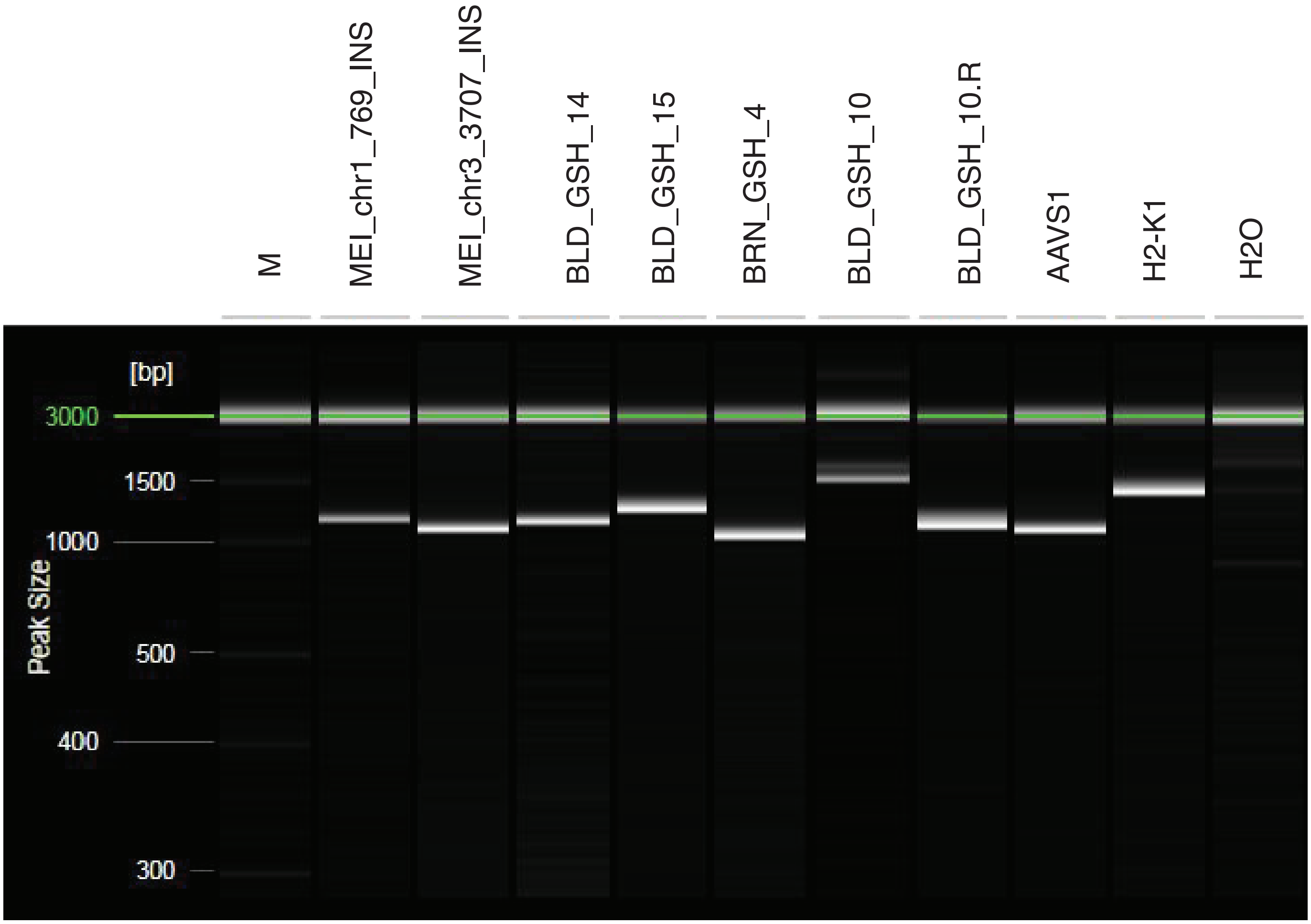

Figure S3: PCR validation of GFP inserted cell line.
The first lane represents the molecular marker, lane 2 and 3 represent GFP integrations in non-GSH MEI sites, lane 4-9 represent GFP integrations in GSH sites, lane 10 represents GFP positive HUDEP2 cells as a positive control and lane 11 is the negative control (water).

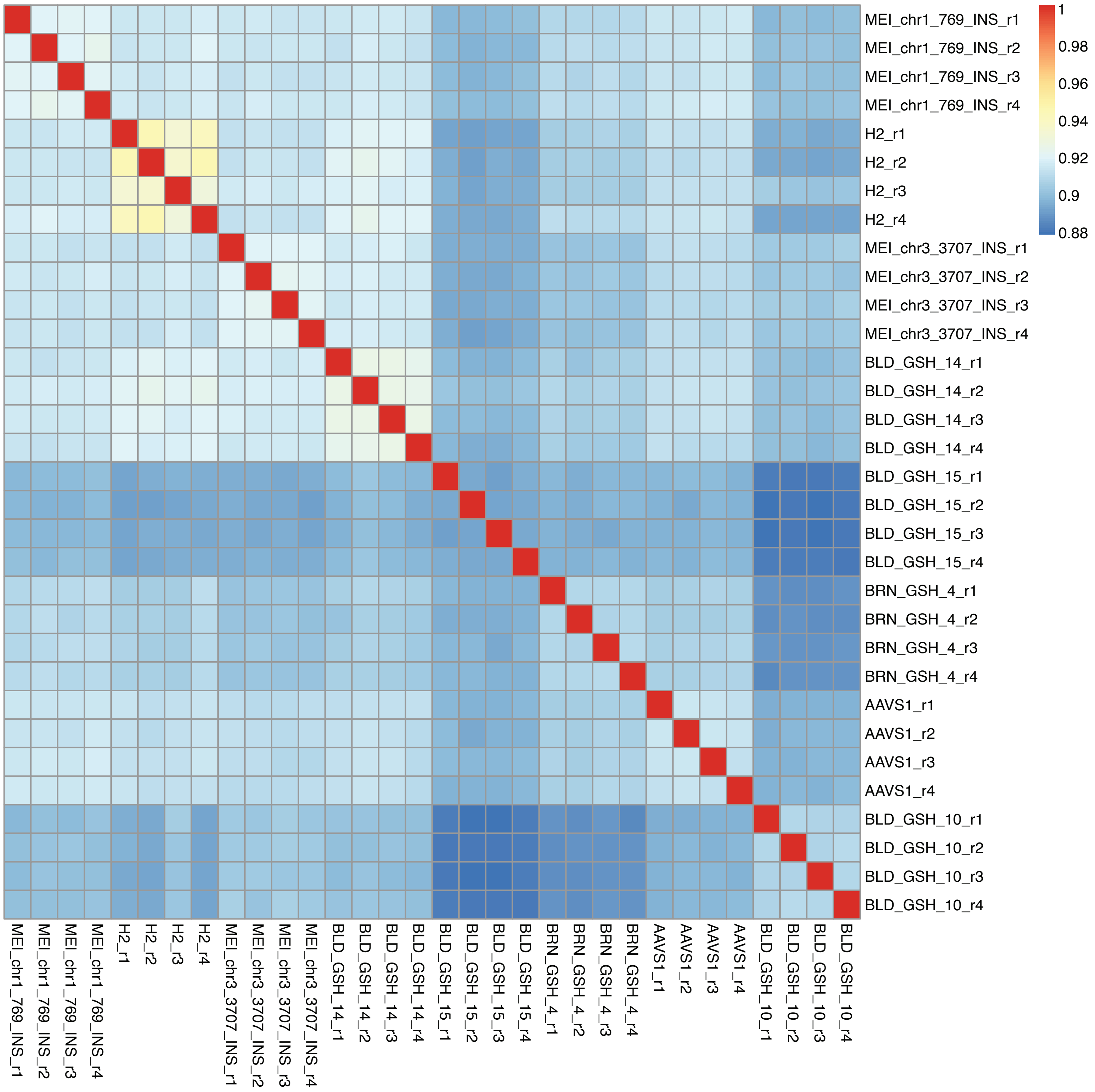

Figure S4: Genome wide gene expression Correlation between GFP integrated cell lines and HUDEP2 cells.
Genome-wide correlation plot between all GFP inserted HUDEP2 cell line and HUDEP2 WT cells.

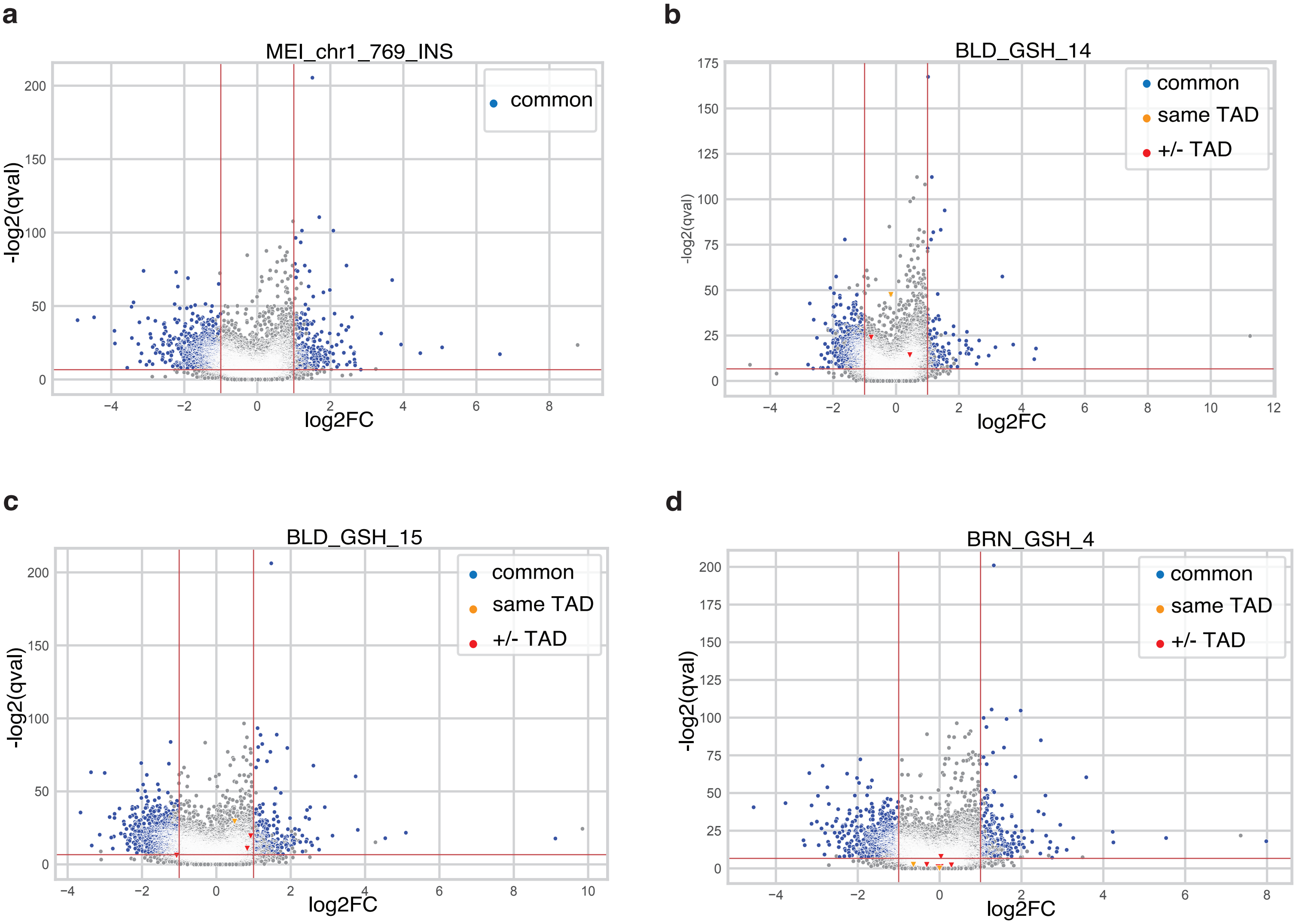

Figure S5: Volcano plot representation of Differentially Expressed Genes for four GSH integration sites. Common: DEGs share by more than two cell lines; Same TAD: genes within the same TAD of the GFP integration site; +/- TAD: genes in the TADs flanking to the GFP integration site.

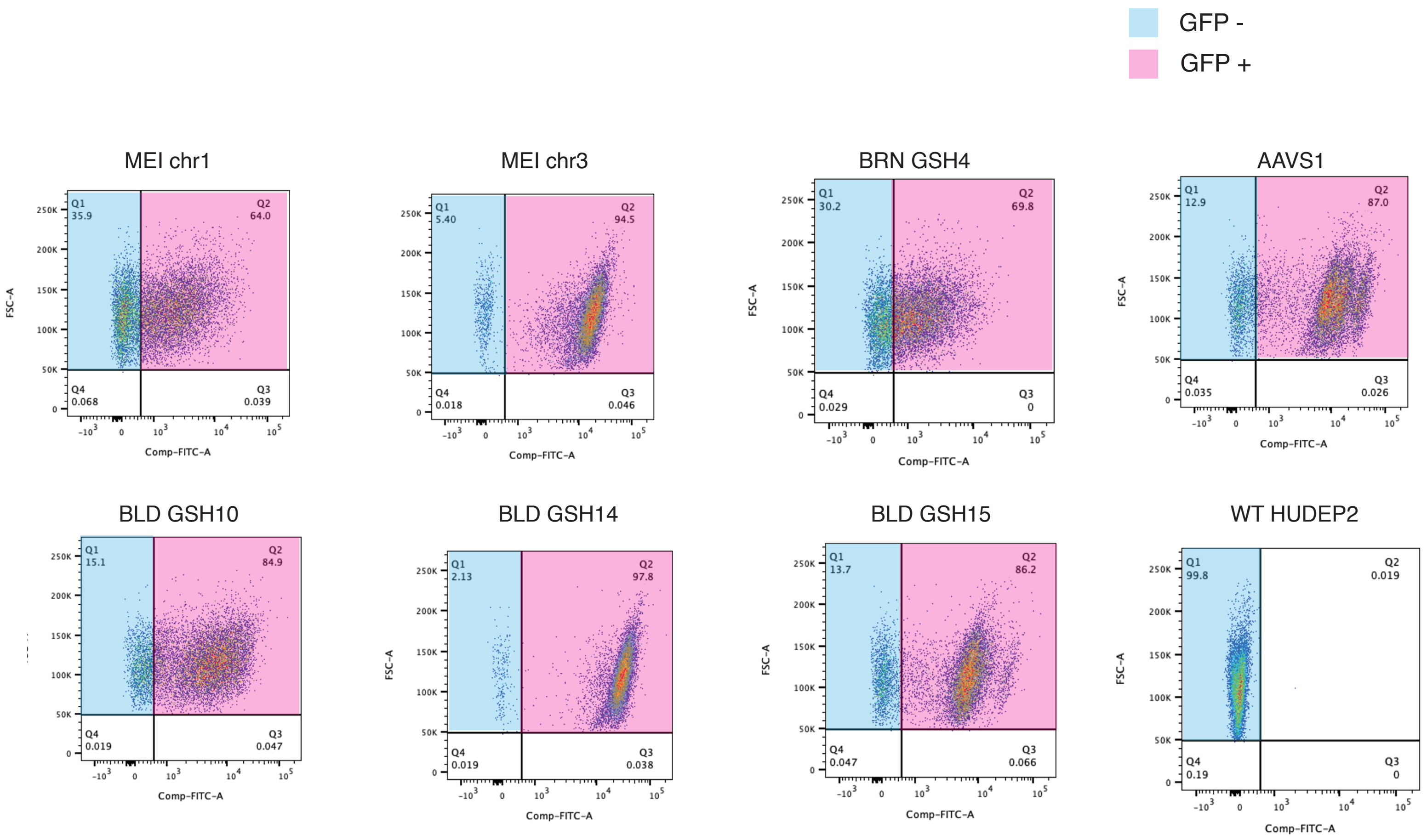

Figure S6: GFP maintenance after one month based on different integration sites.
The GFP expression compartment is highlighted in pink and GFP negative compartment is highlighted in blue.

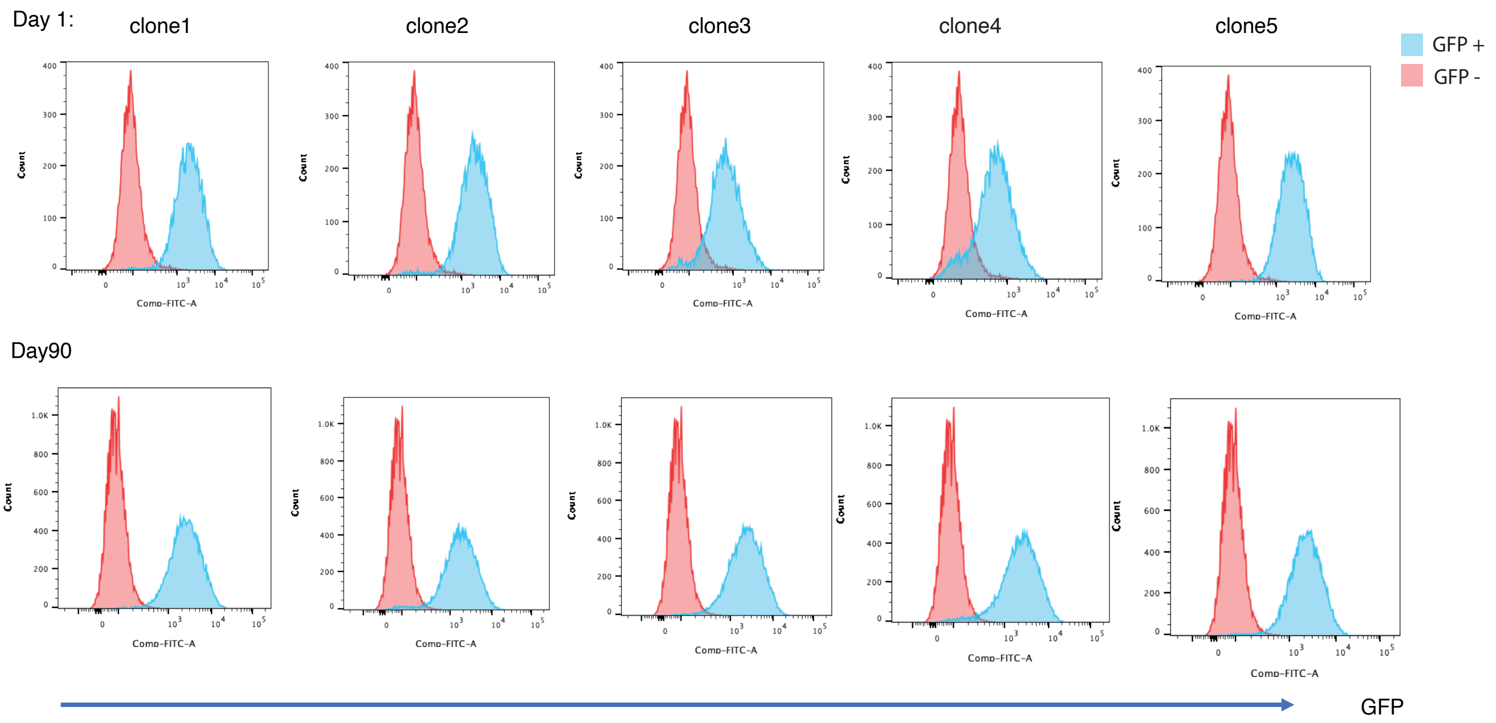

Figure S7: GFP expression in Day 1 and Day 90 for 5 GFP clones of BLD_GSH_10.

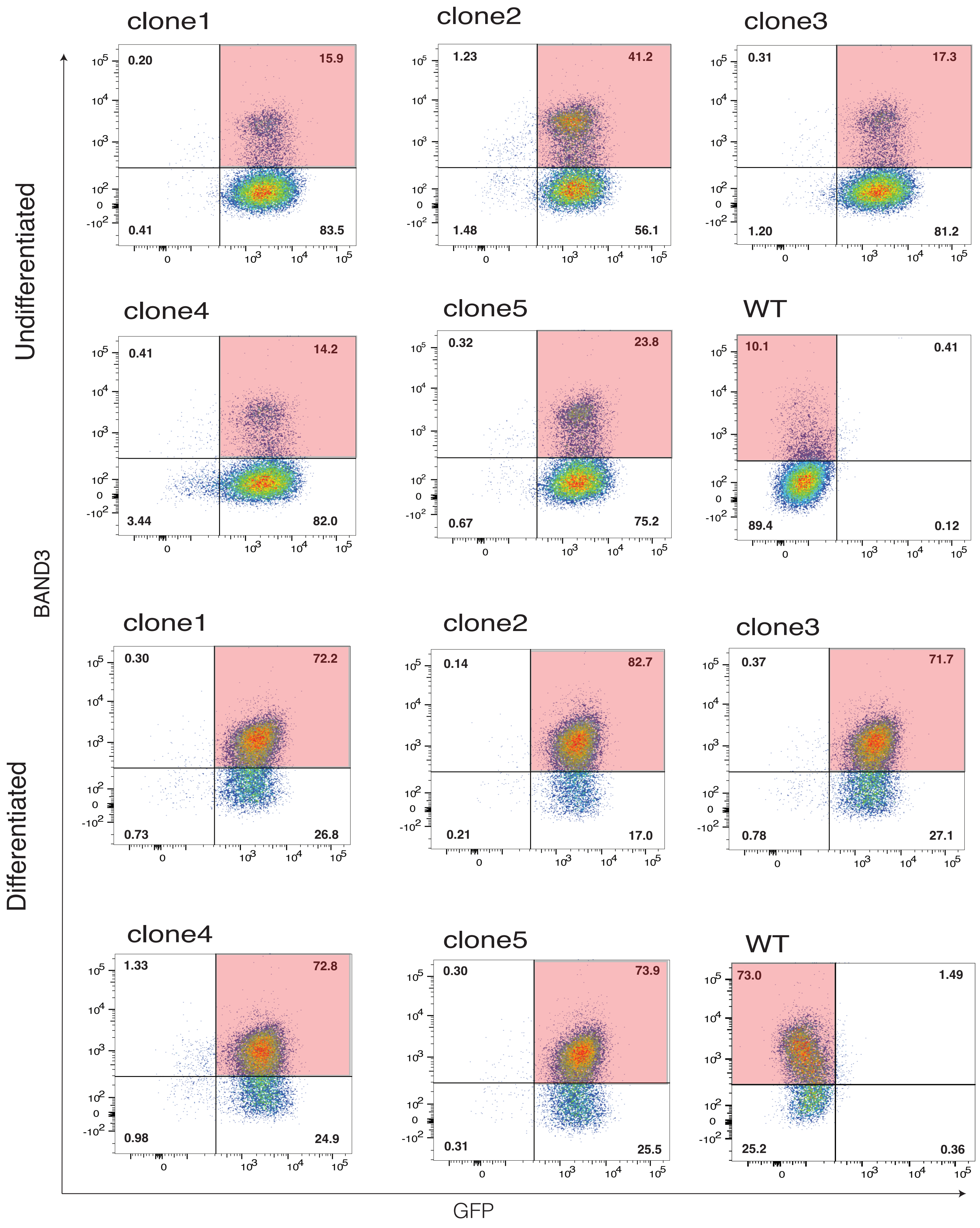

Figure S8: Flow cytometry of GFP and Band3 staining of HUDEP2 GFP clones and HUDEP2 WT cells on day 0 and day 3 of differentiation. The GFP expression compartment is highlighted in red.

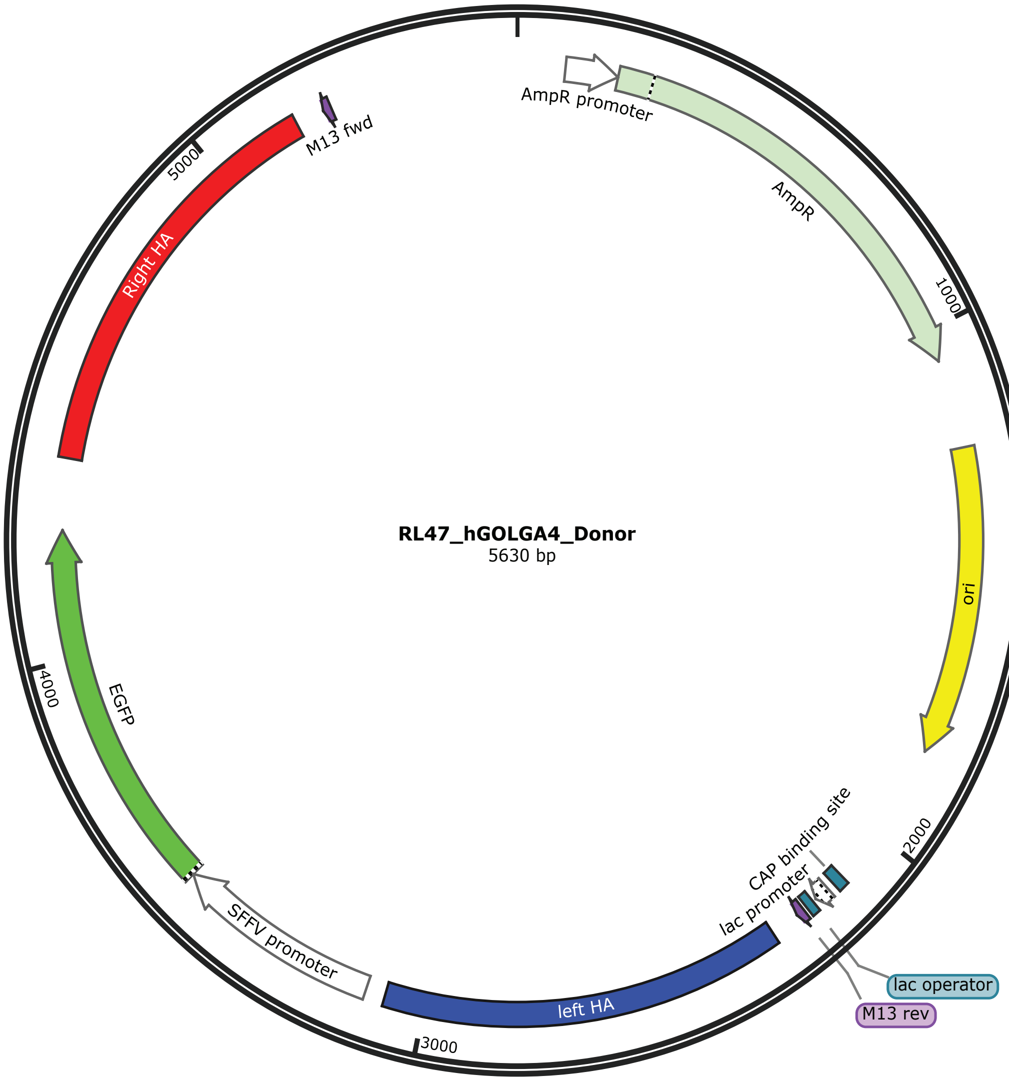

Figure S9: Plasmid vector used for the GFP insertion in HUDEP2 cells.
