# Supplementary material for: Genomics and epigenetics guided identification of tissue-specific genomic safe harbors
Source: Genome Biol. 2022 Sep 21;23:199. doi: 10.1186/s13059-022-02770-3 (PMC9490961; doi:10.1186/s13059-022-02770-3)
Supplement: Supplementary file 10 — Additional file 10. Review history. [file 13059_2022_2770_MOESM10_ESM.docx]

Review History

**First round of review**

**Reviewer 1**

**Are you able to assess all statistics in the manuscript, including the appropriateness of statistical tests used?** Yes, and I have assessed the statistics in my report.

**Comments to author:**

The authors design a framework for identifying genomic safe harbors (GSHs) for genomic editing. To find GSHs, the authors employed 3D chromatin interaction information from Hi-C, polymorphic mobile element insertions (pMEIs) in eQTL studies, and repressive histone mark data. In blood cells, 20 candidate GSHs were identified. The effects of GFP integration into one of the identified GSHs was tested. A program was developed to apply the described framework. A list of 5 brain GSHs were also identified.

This is a very interesting and innovative study. The paper provides a novel framework that could be helpful in gene/cell therapy design with many potential applications. I think it is worth publishing but it needs improvement in rigor. I provide some suggestions to help improve the clarity and rigor of this manuscript.

1. Despite the comprehensive and complex filtering steps, the key step seems to be the repressive marks, which removes 94% candidate pMEIs. This seems to significantly reduce candidate pool. As a result, most candidate GSHs are in introns. The risk of putting an expression cassette in introns should not be underestimated. At least a careful discussion should be provided.
2. What is the orientation of the GFP insertion in GSH_13? Did the authors try both orientations? An analysis of orientation is important since this is an intronic GSH.
3. Gene expression analysis presented in Figure 3 needs a lot more rigor.
a. First, it is unknown whether the wild-type replicates refer to technical replicates or biological replicates. The wild-type replicates should be clones that did not take up the GFP insertion but underwent the clonal selection procedure to provide a meaningful comparison.
b. Second, the correlation between WT replicates and between clones were 0.98, 0.99, whereas correlation between WT and engineered clones were around 0.83. This difference is not small and should not be brushed off. In fact, I suspect the authors had been looking at gene expression variation resulted from the clonal selection process. At least the authors need to establish that there weren't statistically significant differences. A genome-wide differential expression analysis must be presented. Fig 3e alone is not sufficient.
c. Third, please specifically analyze and show candidate genes around the insertion. For example, what is the expression level of GOLGA4? This needs to be highlighted. Please show expression changes of all genes in the upstream/downstream TADs, and compare the pattern to genome-wide patten.
d. Fourth, Fig 3c shows a fair amount of differences but glossed over in text as indifferent. Please use a statistical test wherever "difference" or "no difference" is claimed. Same applies to Fig 3d and several other figures and paragraphs throughout.
4. Key controls are missing. Positive control: effect of GFP in known GSH; background control: GFP should be integrated into several non-GSH regions using the same procedure, and stability of GFP expression and genome-wide gene expression should be compared. Significant work needs to be done to fully address this point; I think the authors should at least provide a thorough discussion of the limitation of current work.
5. Several points need to be discussed, even though I do not require change of analysis strategy or additional experimental support: 1) Tissue-specific eQTL analysis only included protein-coding gene. This leaves out noncoding genes that could also be functionally important; 2) 3D genome interactions all came from the cell line GM12878. These transformed cells could carry different 3D genome structures comparing to blood cells in healthy subjects. 3) transgene-genome interaction could be transgene-dependent, with inserted sequences providing potential inducer for local epigenetic changes and new sites for interactions.

**Reviewer 2**

**Are you able to assess all statistics in the manuscript, including the appropriateness of statistical tests used?** Yes, and I have assessed the statistics in my report.

**Comments to author:**

In this manuscript entitled "Genomics and epigenetics guided identification of tissue-specific genomic safe harbors", Dr. Yong Cheng and colleagues developed a new framework to identify genomic safe harbors (GSHs) based on the available database such as the 1000 Genomes project and GTEx project. They developed a user-friendly pipeline (GEG-SH mapper) for identifying GSH sites. They also found that the GSHs identified in brain and blood cells were unique to each other, highlighting the importance of tissue-specific epigenetic information in GSH identification. This study provides a novel conceptual framework, which has its value. However, this study suffers from multiple defects

Identifying genomic safe harbors have been a long-sought-after goal in gene therapy. There are likely thousands, if not millions, of GSHs in the human genome. Over the last decades, a treasure trove of lentivirus integration sites has been published, and only a small proportion led to clonal proliferation foreboding leukemic transformation. This study, using omics tools, identified a bunch of GSHs of potential interest. However, only one site was supported with experimental data. It is unclear the false positive and false negative rates of their predictions. To make a sound conclusion, at least 3 putative "safe" sites and a couple of putative "unsafe" loci as control should be validated.

For the one confirmed site, the authors need to analyze the whole transcript of GOLGA4. It is unclear whether integration of the SFFV-GFP cassette will affect GOLGA4 expression and splicing.

For gene editing, the HDR donor carries a strong promoter SFFV, which will lead to GFP expression even with random integration. The authors did not show data to rule out this possibility in the selected clones. In one clone, the GFP level was abnormally high on Day 90. The authors need to investigate random integrations.

The GSHs in erythroid cells are not clinically relevant for the following reasons: 1) GOLGA4 expression levels in hematopoietic cells are low, suggestive of lower chromatin accessibility. This will likely lead to lower HDR editing efficiency. Unfortunately, the authors did not report the HDR efficiency. 2) In the gene therapy for red blood cell disorders, one needs to edit hematopoietic stem cells instead of erythroid progenitors. As such, one must study HSC editing as well.

Others:

"Table S1: GFP flow data of day 1 and day 90 for WT and GFP inserted HUDEP2 clones." Better to present the FACS panels.

The 5 data points from 5 clones shown in Figure 3b were not consistent with the legend "three independent clones", and the statistical method was not indicated clearly.

**Authors Response**

**Point-by-point responses to the reviewers’ comments:**

General response:

We thank the reviewers and the editor for their thoughtful comments and suggestions. One major comment from both reviewers was that we only showed validation experiment for one GSH site in the initial submission. In the revised manuscript, we experimentally tested 6 additional loci and brought the total number of tested loci to 7 (3 blood GSHs + 1 brain GSH in the heterochromatin region in blood cells + 2 randomly selected non-GSH polymorphic mobile insertion sites (pMEIs) as negative controls + AAVS1 locus as a positive control). Altogether, we added 28 RNA-seq data with four replicates per cell line. We also performed other analysis suggested by the reviewers to improve the overall rigor. By addressing all the concerns and making the changes recommended, we feel that the revised manuscript is much improved. Major changes are highlighted within the manuscript. Please see below, in blue, for point-by-point responses to the reviewers’ comments and concerns.

Reviewer reports:

Reviewer #1: The authors design a framework for identifying genomic safe harbors (GSHs) for genomic editing. To find GSHs, the authors employed 3D chromatin interaction information from Hi-C, polymorphic mobile element insertions (pMEIs) in eQTL studies, and repressive histone mark data. In blood cells, 20 candidate GSHs were identified. The effects of GFP integration into one of the identified GSHs was tested. A program was developed to apply the described framework. A list of 5 brain GSHs were also identified.

This is a very interesting and innovative study. The paper provides a novel framework that could be helpful in gene/cell therapy design with many potential applications. I think it is worth publishing but it needs improvement in rigor. I provide some suggestions to help improve the clarity and rigor of this manuscript.

1. 	Despite the comprehensive and complex filtering steps, the key step seems to be the repressive marks, which removes 94% candidate pMEIs. This seems to significantly reduce candidate pool. As a result, most candidate GSHs are in introns. The risk of putting an expression cassette in introns should not be underestimated. At least a careful discussion should be provided.

Response: We agree with the reviewer that putting expression cassettes in introns has potential risks. Following the reviewer’s suggestion, we have performed several analyses to evaluate those risks. First, we assessed the risk of alternative splicing due to the GFP integration. We compared the splicing isoforms between wild-type HUDEP2 cells and HUDEP2 cells with integrated expression cassettes using rMATS [1]. There are no significant (FDR<0.01) alternative splicing events detected (Table S4). We also expanded similar analysis to other loci tested and did not detect any significant alternative splicing events either. Thus, at least in our system, cassette integration in introns does not significantly alter the RNA splicing of host genes. Nevertheless, we cannot completely exclude the possibility that other expression cassettes, especially those with splicing acceptor consensus sequences, could affect the nearby gene splicing.

Second, we assessed the risk of transcriptional leakage by examining the RNA-seq signals downstream of integrated GFP cassettes. Compared with wild type HUDEP2, we did observe that almost all GFP integrated sites show different levels of transcripts that extend about 600bp downstream of GFP cassettes. Since the average distance between GSH sites to the nearest exons is 3kb, we did not find transcriptional leakage affect the expression levels of host genes.

We have added these results in the updated “Validation of GSH site in HUDEP2 cells” section. We also added the following discussion in the revised manuscript to describe our results and discuss the potential risk and solution.

“One safety concern of gene therapy is that the integration of gene expression cassettes can potentially change RNA splicing of host genes [39]. Even though no significant alternative splicing events were detected in our study, we cannot completely exclude the possibility for other expression cassettes, especially those with splicing acceptor consensus sequences, could affect the nearby gene splicing. Thus, carefully assessing the splicing events is important for new expression cassettes. Another concern is the transcriptional leakage of gene expression cassettes [40]. In our validation experiment, we observed transcriptional leakage that can extend up to 600 bps downstream of GFP cassettes. One potential solution is adding insulator elements to the cassettes [41].”

2. 	What is the orientation of the GFP insertion in GSH_13? Did the authors try both orientations? An analysis of orientation is important since this is an intronic GSH.

Response: We agree with the reviewer that the direction of the cassette could have an impact on the nearby gene expression. In the GSH_13 (BLD_GSH_10 in our revised manuscript) locus, the orientation of the *GFP* gene in our initial cassette is the same as *GOLGA4*. To assess the effect of the cassette orientation, we generated another cell line with *GFP* integrated in the reverse direction. We compared RNA-seq data between cells with opposite *GFP* integration directions and found no significant difference in expression levels (LFC=-0.2, FDR=2.5E-04) and no changes in RNA splicing (FDR<0.01, rMATS) of *GOLGA4*.  We have added the orientation analysis in the revised manuscript.

“We further flipped the orientation of the GFP cassette at the GOLGA4 locus and found that neither direction affects the splicing of *GOLGA4* (Table S4). In addition, the expression levels of *GOLGA4* and other genes within the same TAD did not change significantly (FDR< 0.01, LFC>1 or LFC <-1) between the two cassette integration directions. ”

3. 	Gene expression analysis presented in Figure 3 needs a lot more rigor.

a. 	First, it is unknown whether the wild-type replicates refer to technical replicates or biological replicates. The wild-type replicates should be clones that did not take up the GFP insertion but underwent the clonal selection procedure to provide a meaningful comparison.

Response: We thank the reviewer for this thoughtful suggestion. The wild-type replicates are biological replicates with RNA prepared from three different preparations. They are bulk cells without clonal selection procedure. Following the reviewer’s suggestion, we generated three wild-type Hudep2 clones. The correlation coefficient among the three newly generated wild type HUDEP2 clones are 0.82, 0.83 and 0.81, which are similar to those among GFP clones and less than those among bulk HUDEP2 cells. To avoid this clonal variation, for RNA-seq comparison in the revised manuscript, we used bulk sorted GFP integration cell lines with bulk Hudep2 cells as control. For each cell line, RNAs were prepared from four batches of bulk sorted GFP cells as biological replicates. We have added the information to the revised method section.

b. 	Second, the correlation between WT replicates and between clones were 0.98, 0.99, whereas correlation between WT and engineered clones were around 0.83. This difference is not small and should not be brushed off. In fact, I suspect the authors had been looking at gene expression variation resulting from the clonal selection process. At least the authors need to establish that there weren't statistically significant differences. A genome-wide differential expression analysis must be presented. Fig 3e alone is not sufficient.

Response: As the reviewer hypothesized, the differences among engineered clones are largely due to clonal variation. The correlation coefficient among the three newly generated wild type HUDEP2 clones are 0.82, 0.83 and 0.81, similar to those among GFP clones.

In the revised manuscript, we performed differential expression analysis at genome-wide level for all the GFP integrated cell lines established.  As the reviewer pointed out, integration of a GFP cassette, regardless of the integration site, induced a global expression change of ~1,000 genes. Most ( ~80%) of these differential expressed genes are shared among at least three cell lines with different integration sites. Gene ontology (GO) enrichment analysis showed that genes involved protein degradation such Ubl conjugation are highly enriched (FDR=1.1E-8), indicating that the expression level changes of those genes are likely triggered by cellular response to GFP [2,3]. We have added the genome-wide differential expression analysis in the “Validation of GSH site in HUDEP2 cells” section.

c. 	Third, please specifically analyze and show candidate genes around the insertion. For example, what is the expression level of GOLGA4? This needs to be highlighted. Please show expression changes of all genes in the upstream/downstream TADs, and compare the pattern to genome-wide patten.

Response: We thank the review for this thoughtful suggestion. In the revised manuscript, we analyzed the expression level changes for *GOLGA4* and other genes within the same TAD and flanking TADs. We also compared the local pattern to the genome-wide pattern for all the tested loci. In all three GSHs tested, there are no significantly changed (sleuth [4], FDR<0.01, LFC >1 or < -1) genes within the same TAD and flanking TADs of the GSH. In contrast, the GFP cassette integrated into one randomly selected MEI (MEI_chr3_3707_INS) lead to significantly increased expression of *PTX3* (LFC=1.12, FDR=9.39E-05). GFP integrated in the *AAVS1* locus also alternated the expression of two genes, *TNNI3* (LFC=1.68, FDR=3.17E-04) and *PPP6R1* (LFC=-1.59, FDR=1.34E-07).

We reported this result in the revised “Validation of GSH site in HUDEP2 cells” section.

Fig 4c. Representative immuno-flow cytometry results showing cell differentiation comparison between WT cells and cells from one HUDEP2 GFP clone. Y-axis is the signal for red blood cell maturation marker Band3. X-axis is the signals for GFP. The mature red blood cell compartment is highlighted in red.

d. 	Fourth, Fig 3c shows a fair amount of differences but glossed over in text as indifferent. Please use a statistical test wherever "difference" or "no difference" is claimed. Same applies to Fig 3d and several other figures and paragraphs throughout.

Response: We apologies for the lack of clarity. In Fig3c, the matured red blood cells were GFP

positive and Band3 positive in GFP integrated cells. In contrast, for WT HUDEP2 cells, they are GFP negative and Band3 positive. Thus, after differentiation, there are 72.2% mature red blood cells in GFP integrated cells which is close to the 73% in WT HUDEP2 cells. We have updated the Fig 4c (shown left) by highlighting the mature red blood cell compartment.

In addition, we added detailed statistical tests information for all "difference" or "no difference" mentioned in the manuscript.

4. 	Key controls are missing. Positive control: effect of GFP in known GSH; background control: GFP should be integrated into several non-GSH regions using the same procedure, and stability of GFP expression and genome-wide gene expression should be compared. Significant work needs to be done to fully address this point; I think the authors should at least provide a thorough discussion of the limitations of current work.

Response: As the reviewer suggested, we have tested two additional blood GSHs in the revised manuscript. We also included two randomly selected MEIs, one brain GSH located in heterochromatin in blood cells as negative controls and the AAVS1 locus as a positive control. We performed RNA-seq and compared the expression profiles among all cell lines. Since there are global expression changes after delivering the GFP cassette, we focused on genes within the same TAD of integration sites. Among the three blood GSHs, there are no significantly changed genes (FDR<0.01, LFC>1 or LFC < -1). In contrast, the GFP cassette integrated into one randomly selected MEI (MEI_chr3_3707_INS) lead to significantly increased (FC=1.12, FDR=9.39E-05) expression of *PTX3*. GFP integrated in the AAVS1 locus also alternated the expression of two genes, *TNNI3* (LFC=1.68, FDR=3.17E-04) and *PPP6R1* (LFC=-1.59, FDR=1.34E-07).

We have integrated the analysis results in the revised “Validation of GSH site in HUDEP2 cells” section.

5. 	Several points need to be discussed, even though I do not require change of analysis strategy or additional experimental support: 1) Tissue-specific eQTL analysis only included protein-coding gene. This leaves out noncoding genes that could also be functionally important; 2) 3D genomeinteractions all came from the cell line GM12878. These transformed cells could carry different 3D genome structures comparing to blood cells in healthy subjects. 3) transgene-genome interaction could be transgene-dependent, with inserted sequences providing potential inducer for local epigenetic changes and new sites for interactions.

Response:

1) eQTL for non-coding genes.

We thank the reviewer for bringing up the distinction between coding and non-coding genes. For the GTEx eQTL analysis, we included both coding and non-coding genes in the initial analysis. On the other hand, for the 1000 Genomes Project data set in blood, we only included protein coding genes in the eQTL analysis. We agree with the reviewer that it is important to include the potential impact of pMEIs on the non-coding gene expression as a part of the selection criteria. In the revised manuscript, we now included non-coding genes in the 1000 Genomes eQTL analysis and rerun the pipeline based on the updated eQTL results. Including non-coding gene eQTLs resulted in four fewer GSH sites from the 1000 Genomes dataset. The total number of GSH derived from the 1000 Genomes dataset is now 16. However, among the nine GSHs, six are shared between the 1000 Genomes and GTEx data, further supporting the confidence of the GSHs. We updated the manuscript to include the description of the new analysis and the revised results.

2) Even though TADs used in our pipeline is a fundamental genomic feature and generally similar among multiple cell types [5], we agree with the reviewer that there can be different 3D genome structures between cell line and primary cells. We added an option in our pipeline to let users use their own functional genomic data including TADs. In addition, we discussed this limit in the revised manuscript.

“Another limit in our pipeline is that some functional genomic data used are from cell lines instead of more clinically relevant primary cells such as CD34+ hematopoietic stem and progenitor cells (HSPCs). This is largely due to the data availability. Our pipeline does have an option to let users provide their own functional genomic data and identify best GSHs for their own systems.”

3) We agree with the reviewer that different transgenes could have specific effects on a given genomic region. This possibility highlighted the need of having extensive validation on the GSH candidate sites for a specific application. The benefit of our approach is to provide a list of potential GSHs, and the researcher can perform further tests to select the GSH sites that work the best for their specific transgenes. We added the following text in the Discussion section:

“We used a GFP expression cassette in our experimental validation. Since transgene-genome interaction can be transgene-dependent, expression cassettes with different transgene can potentially induce different local epigenetic and chromatin interactions changes. Thus, researchers should perform their own tests to select the GSHs that work the best for their specific studies.”

Reviewer #2: In this manuscript entitled "Genomics and epigenetics guided identification of tissue-specific genomic safe harbors", Dr. Yong Cheng and colleagues developed a new framework to identify genomic safe harbors (GSHs) based on the available database such as the 1000 Genomes project and GTEx project. They developed a user-friendly pipeline (GEG-SH mapper) for identifying GSH sites. They also found that the GSHs identified in brain and blood cells were unique to each other, highlighting the importance of tissue-specific epigenetic information in GSH identification. This study provides a novel conceptual framework, which has its value. However, this study suffers from multiple defects

Identifying genomic safe harbors have been a long-sought-after goal in gene therapy. There are likely thousands, if not millions, of GSHs in the human genome. Over the last decades, a treasure trove of lentivirus integration sites has been published, and only a small proportion led to clonal proliferation foreboding leukemic transformation. This study, using omics tools, identified a bunch of GSHs of potential interest. However, only one site was supported with experimental data. It is unclear the false positive and false negative rates of their predictions. To make a sound conclusion, at least 3 putative "safe" sites and a couple of putative "unsafe" loci as control should be validated.

Response: As both reviewers suggested, we have tested more loci in the revised manuscript. Specifically, we included two additional blood GSHs. We also added two randomly selected non-GSH MEIs and one brain GSH located within heterochromatin in blood cells as negative controls, and the AAVS1 locus was also added as a positive control. We performed RNA-seq and GFP expression analysis on the new cell lines. Altogether, we added 28 RNA-seq data with four replicates per cell line. We also performed other analysis suggested by the reviewers to improve the overall rigor.

We have integrated the analysis results in the revised “Validation of GSH site in HUDEP2 cells” section.

For the one confirmed site, the authors need to analyze the whole transcript of GOLGA4. It is unclear whether integration of the SFFV-GFP cassette will affect GOLGA4 expression and splicing.

Response: We thank the reviewer for this thoughtful suggestion. In the revised manuscript, we analyzed the expression level changes (Kallisto and Sleuth) [4,6] of genes within the same TAD of GFP integration sites. Among the three blood GSHs, there are no significantly changed genes (FDR<0.01, LFC>1 or LFC<-1). We also compared RNA splicing between wild-type HUDEP2 and HUDEP2 cells with GFP integration with rMATS and did not find any significant difference (FDR<0.01).

We have integrated the analysis results in the revised “Validation of GSH site in HUDEP2 cells” section.

For gene editing, the HDR donor carries a strong promoter SFFV, which will lead to GFP expression even with random integration. The authors did not show data to rule out this possibility in the selected clones. In one clone, the GFP level was abnormally high on Day 90. The authors need to investigate random integrations.

Vector copy number in each clone estimated by qPCR.

Response: We thank the reviewer for raising this important question. We measured vector copy number per cell by quantitative polymerase chain reaction (qPCR) with the use of a standard curve derived from a previously characterized cell clone with one GFP integration site (by FISH and the quantitative shearing linear amplification PCR method [7]). The result indicated that all the clones have only one GFP cassette when initially cultured (shown left). The high GFP level in one clone may be due to the clonal variation during long-term cell culture.

The GSHs in erythroid cells are not clinically relevant for the following reasons: 1) GOLGA4 expression levels in hematopoietic cells are low, suggestive of lower chromatin accessibility. This will likely lead to lower HDR editing efficiency. Unfortunately, the authors did not report the HDR efficiency. 2) In the gene therapy for red blood cell disorders, one needs to edit hematopoietic stem cells instead of erythroid progenitors. As such, one must study HSC editing as well.

Response: We agree with the reviewer that primary cells such as CD34+ HSPC or T-cells are more clinically relevant. However, the main benefit of our study is to provide a list of potential GSH sites. For any clinically related studies, researchers need to perform more experiments to optimize the editing efficiency and evaluate long-term safety.  In addition, we also provide a program that users can apply the same framework to identify candidate GHSs in their own cell system.

In the HUDEP2 system, the average CRISPR gRNA efficiency is more than 60% and the HDR editing efficiency is usually close to 3-5%. As the reviewer mentioned, we expect the efficiency in primary cells are likely even lower. In fact, low efficiency of homology-directed repair remains a major hurdle in precise gene therapy. We do anticipate that by chemically and genetically favoring the HDR pathway during DNA repair [8], improving Cas9 delivery system [9], modifying cas9 [10,11], and developing new genome editing technology [12], the HDR efficiency will dramatically increase in the future.

We also added the limits of using cell lines in the discussion.

“Another limit in our pipeline is that some functional genomic data used are from cell lines instead of more clinically relevant primary cells such as CD34+ hematopoietic stem and progenitor cells (HSPCs). This is largely due to the data availability. Our pipeline does have an option to let users provide their own functional genomic data and identify the best GSHs for their own systems.”

Others:

"Table S1: GFP flow data of day 1 and day 90 for WT and GFP inserted HUDEP2 clones." Better to present the FACS panels.

Response: We apologize for the lack of clarity. The FACS panels are now included in the supplementary file Figure S6.

The 5 data points from 5 clones shown in Figure 3b were not consistent with the legend "three independent clones", and the statistical method was not indicated clearly.

Figure 4b: Bar plots showing the normalized GFP fluorescence signals of five independent clones and wild type HUDEP2 cells.

Response: We apologize for the lack of clarity. The FACS data were based on 5 clones. We have updated the legend.

We compared the GFP levels between clones and wild-type HUDEP2 cells and found GFP expression levels were well- maintained in both Day0 and Day90. Since the GFP level in WT HUDEP2 is close to 0. We did not use any statistic test. To avoid confusion, we added GFP level of the WT HUDEP2 cells in Fig 4b (shown left).

Reference

1. Shen S, Park JW, Lu Z, Lin L, Henry MD, Wu YN, et al. rMATS: robust and flexible detection of differential alternative splicing from replicate RNA-Seq data. Proc Natl Acad Sci U S A. 2014;111:E5593-601.

2. Coumans JVF, Gau D, Poljak A, Wasinger V, Roy P, Moens P. Green fluorescent protein expression triggers proteome changes in breast cancer cells. Exp Cell Res. 2014;320:33–45.

3. Liu HS, Jan MS, Chou CK, Chen PH, Ke NJ. Is green fluorescent protein toxic to the living cells? Biochem Biophys Res Commun. United States; 1999;260:712–7.

4. Pimentel H, Bray NL, Puente S, Melsted P, Pachter L. Differential analysis of RNA-seq incorporating quantification uncertainty. Nat Methods. United States; 2017;14:687–90.

5. Smith EM, Lajoie BR, Jain G, Dekker J. Invariant TAD Boundaries Constrain Cell-Type-Specific Looping Interactions between Promoters and Distal Elements around the CFTR Locus. Am J Hum Genet. 2016;98:185–201.

6. Bray NL, Pimentel H, Melsted P, Pachter L. Near-optimal probabilistic RNA-seq quantification. Nat Biotechnol. 2016;34:525–7.

7. Zhou S, Bonner MA, Wang Y-D, Rapp S, De Ravin SS, Malech HL, et al. Quantitative shearing linear amplification polymerase chain reaction: an improved method for quantifying lentiviral vector insertion sites in transplanted hematopoietic cell systems. Hum Gene Ther Methods. 2015;26:4–12.

8. Maruyama T, Dougan SK, Truttmann MC, Bilate AM, Ingram JR, Ploegh HL. Increasing the efficiency of precise genome editing with CRISPR-Cas9 by inhibition of nonhomologous end joining. Nat Biotechnol. 2015;33:538–42.

9. Yang D, Scavuzzo MA, Chmielowiec J, Sharp R, Bajic A, Borowiak M. Enrichment of G2/M cell cycle phase in human pluripotent stem cells enhances HDR-mediated gene repair with customizable endonucleases. Sci Rep. 2016;6:21264.

10. Charpentier M, Khedher AHY, Menoret S, Brion A, Lamribet K, Dardillac E, et al. CtIP fusion to Cas9 enhances transgene integration by homology-dependent repair. Nat Commun. 2018;9:1133.

11. Hess GT, Tycko J, Yao D, Bassik MC. Methods and Applications of CRISPR-Mediated Base Editing in Eukaryotic Genomes. Mol Cell. 2017;68:26–43.

12. Jiang T, Zhang X-O, Weng Z, Xue W. Deletion and replacement of long genomic sequences using prime editing. Nat Biotechnol. 2022;40:227–34.

**Second round of review**

**Reviewer 1**

The authors have improved the rigor of the study, and clarified the specific contribution of their results to the field. I recommend the publication of this manuscript.

**Reviewer 2**

Satisfied.
